# Atlantic mackerel population structure does not support genetically distinct spawning components

**DOI:** 10.12688/openreseurope.17365.2

**Published:** 2025-01-20

**Authors:** Alice Manuzzi, Imanol Aguirre-Sarabia, Natalia Díaz-Arce, Dorte Bekkevold, Teunis Jansen, Jessica Gomez-Garrido, Tyler S. Alioto, Marta Gut, Martin Castonguay, Sonia Sanchez-Maroño, Paula Álvarez, Naiara Rodriguez-Ezpeleta

**Affiliations:** 1 AZTI, Marine Research, Basque Research and Technology Alliance (BRTA), Sukarrieta, Spain; 2 DTU Aqua, National Institute of Aquatic Resources, Section for Marine Living Resources, Silkeborg, Denmark; 3 GINR, Greenland Institute of Natural Resources, Nuuk, Greenland; 4 Centro Nacional de Análisis Genómico (CNAG), Barcelona, Spain; 5 Universitat de Barcelona (UB), Barcelona, Spain; 6 Maurice Lamontagne Institute, Fisheries and Oceans Canada, Mont-Joli, Canada, Mont-Joli, Canada

**Keywords:** Atlantic mackerel, complete genome, RAD-seq, population structure, genome-wide SNPs, fisheries management

## Abstract

**Background:**

The Atlantic mackerel, *Scomber scombrus* (Linnaeus, 1758) is a commercially valuable migratory pelagic fish inhabiting the northern Atlantic Ocean and the Mediterranean Sea. Given its highly migratory behaviour for feeding and spawning, several studies have been conducted to assess differentiation among spawning components to better define management units, as well as to investigate possible adaptations to comprehend and predict recent range expansion northwards.

**Methods:**

Here, the genome of *S. scombrus* was sequenced and annotated, as an increasing number of population genetic studies have proven the relevance of reference genomes to investigate genomic markers/regions potentially linked to differences at finer scale. Such reference genome was used to map Restriction-site-associated sequencing (RAD-seq) reads for SNP discovery and genotyping in more than 500 samples distributed along the species range. The resulting genotyping tables have been used to perform connectivity and adaptation analyses.

**Results:**

The assembly of the reference genome for *S. scombrus* resulted in a genome of 741 Mb. Our population genetic results show that the Atlantic mackerel consist of three previously known genetically isolated units (Northwest Atlantic, Northeast Atlantic, Mediterranean), and provide no evidence for genetically distinct spawning components within the Northwest or Northeast Atlantic.

**Conclusions:**

Therefore, our findings resolved previous uncertainties by confirming the absence of genetically isolated spawning components in each side of the northern Atlantic, thus rejecting homing behaviour and the need to redefine management boundaries in this species. In addition, no further genetic signs of ongoing adaptation were detected in this species.

## Plain language summary

The Atlantic mackerel is a commercially valuable migratory pelagic fish inhabiting the northern Atlantic Ocean and the Mediterranean Sea. In both sides of the Atlantic, this species spawns at several locations (spawning components) while migrating north for feeding. It has been hypothesized that the mackerel has a homing behaviour, meaning that they return to spawn to where they were born, in which case, genetic differences should be visible between spawning components. Here, we have sequenced the complete genome of Atlantic mackerel and performed population genetic analyses based on thousands of markers. Our results provide no evidence for genetically distinct spawning components which implies that, for fisheries management, this species should be managed as one unit in each side of the Atlantic.

## Introduction

The Atlantic mackerel, *Scomber scombrus* L., is a highly migratory coastal pelagic fish widely distributed throughout the northern Atlantic Ocean, from Labrador, Canada, to Cape Lookout, United States, in the west, and from Iceland and Norway to as far south as Mauritania in the east, as well as in the Mediterranean Sea (MED) and in the Black Sea ( Iversen, 2002; Lockwood, 1988; Trenkel *et al.*, 2014). Within the northwest Atlantic (NWA), two spawning components have been recognized: a southern component, spawning along the US East coast in March/April, and a northern component spawning around the Gulf of St. Lawrence in June/July ( Grégoire *et al.*, 2010; O’Brien *et al.*, 1993; Sette, 1950). Within the northeast Atlantic (NEA), three spawning components have been defined: a southern component, spanning at the Cantabrian Sea and Atlantic Iberian waters, a western component, including the Bay of Biscay, Celtic Seas and West of Scotland and a North Sea component, including the North Sea, Skagerrak and Kattegat, with spawning starting in February off Portugal, and ending in July north of Scotland. Summer northward feeding migrations occur on both sides of the Atlantic ( Godø *et al.*, 2004; Holst & Iversen, 1992; Nesbø *et al.*, 2000; Radlinski *et al.*, 2013; Studholme, 1999; Uriarte & Lucio, 2001).

In the past two decades distribution of Atlantic mackerel has shifted poleward, increasing abundance around Iceland, Greenland, and the Faroe Islands ( Nøttestad *et al.*, 2016). As a consequence of this this climate change-related expansion ( Jansen *et al.*, 2016), the resulting disagreement over quotas escalated into the 'mackerel crisis' ( Jensen *et al.*, 2015), so called for having exacerbated relations among the countries involved. This poleward expansion has been associated to changing water temperatures and food availability ( Jansen *et al.*, 2012; Olafsdottir *et al.*, 2019; Overholtz *et al.*, 2011; Payne *et al.*, 2022), environmental conditions affecting interannual changes in post- and pre-spawning patterns and migration timing ( Jansen & Gislason 2011; Trenkel *et al.*, 2014). In fact, a recent study forecasted a 370km northward shift in mackerel spawning distribution for every degree of sea temperature increase ( Chust *et al.*, 2023).

The Atlantic mackerel holds significant economic importance for numerous countries harvesting the species ( Jensen *et al.*, 2015); only in the NEA, catches of 1084 thousand tonnes ( ICES, 2023) are reported for the species in the ICES areas, corresponding to approximately 1048 million € in sales value (mean yearly value per kg from EU sale; https://eumofa.eu/first-sale-monthly-data). In the NEA, ICES recognizes the existence of different spawning components within a unique stock. Similarly, the species is also considered part of a single stock in the NWA but, despite a collaborative effort, assessment and management are performed independently by the United States and Canada ( DFO, 2021; NEFSC, 2018). Consequently, the Atlantic mackerel has been the subject of numerous studies aimed at understanding the connectivity within its range, especially regarding the migratory dynamics (spawning, feeding, overwintering) of the species that may contribute to population structure and require consideration in fishery assessment. Despite high dispersal rates suggesting potentially high connectivity throughout the species’ range ( Lockwood, 1988; Uriarte & Lucio, 2001; Uriarte *et al.*, 2001), several studies have confirmed the division between NWA and NEA mackerel ( Gíslason *et al.*, 2020; Nesbø *et al.*, 2000; Rodriguez-Ezpeleta *et al.*, 2016), yet contrasting results still exist for both sides of the Atlantic on the existence of genetic differentiation linked to spawning contingents ( Gíslason *et al.*, 2020; Jansen & Gislason, 2013; NEFSC, 2018; Uriarte *et al.*, 2001).

It has been hypothesized that the two NWA components show fidelity to their respective spawning sites ( Studholme, 1999), and studies based on tagging and seasonal landing patterns further suggest that the individuals from the different components undergo distinct seasonal migrations ( Beckett *et al.*, 1974; Sette, 1950). While one recent study based on otolith chemistry appears to have moderate discriminatory power ( Arai *et al.*, 2021), other studies using meristic markers ( Mackay & Garside, 1969), otolith shape ( Castonguay *et al.*, 1991) as well as genetic markers from allozymes ( Maguire *et al.*, 1987), mitochondrial DNA markers ( Lambrey de Souza *et al.*, 2006) and microsatellites ( Gíslason *et al.*, 2020) have failed to discriminate between spawning components. Nevertheless, a recent genetic study based on a higher number of genome-wide markers (single nucleotide polymorphisms; SNPs) has been able to perform individual genetic assignment to component of origin, despite the lack of a clear genetic population differentiation ( Bourret *et al.*, 2023). Likewise, weak spawning site fidelity has been indicted for the NEA based on growth data ( Jansen *et al.*, 2013) while additional studies in support of this hypothesis based on alternative methodologies (e.g. otoliths; Dawson (1991), and parasite infections; Somdal and Schram (1992)) were found inconclusive due to time-space biased sampling. Mixing of the components occurs, as demonstrated by tagging studies where the same mature individuals were detected in more than one component during spawning season ( Uriarte *et al.*, 2001), pointing towards the hypothesis of a panmictic population ( Jansen & Gislason, 2013). Originally, site fidelity was proposed based on the analyses of two mitochondrial DNA regions ( Nesbø *et al.*, 2000). However, this analysis was based on relatively few individuals and it did not account for differences between year classes ( Jansen & Gislason, 2013). Another genetic analysis based on microsatellites did not find any support for separation between spawning components ( Gíslason *et al.*, 2020), suggesting lack of homing behaviour. This lack of a clear differentiation, coupled with the highly migratory behaviour of the species, may indicate spawning in the NEA could occur within a single large spatiotemporal continuum, being close to panmictic but with a weak dynamic cline rather than distinct components ( Jansen & Gislason, 2013).

Given the economic value of the Atlantic mackerel and the recent changes in its distribution, the need to resolve remaining doubts about its connectivity has become urgent. Genomic approaches can provide valuable information to answer the questions presented so far, even more so if a reference genome is used ( Lu & Luo, 2020; Stapley *et al.*, 2010). Recently, there have been major developments in the production of accessible reference genomes ( Fan *et al.*, 2020) as they are recognised as a key tool for improving conservation and management approaches ( Hohenlohe *et al.*, 2021; Rhie *et al.*, 2021; Thorburn *et al.*, 2023). Indeed, the use of species-specific genomes might help to answer fundamental questions about genetic diversity, population structure or the genetic basis of adaptation ( Fuentes-Pardo & Ruzzante, 2017; Whitacre *et al.*, 2022), for instance by identifying regions under selection due to environmental change ( Merot *et al.*, 2023), and improves the feasibility of studies monitoring and predicting effects of climate change. This type of studies may be of great relevance for marine species, as marine diversity is particularly vulnerable to climate change ( Cheung *et al.*, 2009) and have proven to be relevant for fisheries management and conservation ( Ovenden *et al.*, 2015).

Here, we present a high-quality scaffold-level reference genome for Atlantic mackerel, as well as the first population genetic study using reference-based genome-wide markers and across most of the distribution range of the species. Single nucleotide polymorphisms (SNPs) were obtained from samples collected throughout the species whole range, including recent areas of expansion (e.g. Greenland), in order to: (1) assess fine-scale population structure and connectivity existing within the genetically distinct NWA, NEA and MED populations; (2) investigate the origin of the Greenlandic/Icelandic mackerel and possible adaptive markers associated with the recent northward migration; and finally (3) evaluate the possible link between spawning components and genetics by identifying markers that could be used to estimate components proportions within management units.

## Methods

### Tissue sampling and nucleotide extraction

*S. scombrus* larvae, juvenile as well as spawning and non-spawning and adult samples were collected from Northwest (NWA) and Northeast Atlantic Ocean (NEA) and Mediterranean Sea (MED) locations using scientific surveys and commercial fisheries (Supplementary Table S1 in *Extended data* ( Manuzzi *et al.*, 2024); Figure 2.A). Larvae were collected using Bongo 40 or Gulf VII plankton nets and stored in 96% molecular grade ethanol at -20°C. Likewise, from each individual a ~1 cm ^3^ muscle tissue piece was excised and immediately stored in 96% molecular grade ethanol at -20°C until DNA extraction. Maturity of samples was assigned according to six scale categories ( Walsh & Hopkins, 1990). For one female adult individual captured in the northeastern Atlantic (ICES Area VIIj2), muscle, gill, heart and gonad tissues were excised and stored in RNAlater™ (Ambion) until RNA extraction and was used for genome assembly and annotation. DNA was extracted from about 20 mg of muscle tissue or whole larvae using the Wizard® Genomic DNA Purification kit (cat # A1120, Promega, WI, USA) following the manufacturer’s instructions. Extracted DNA was suspended in Milli-Q® IQ 7000 (Millipore) water, 20 µl in the case of larvae and 100 µl for tissues. Concentration was determined with the Quant-iT dsDNA HS assay kit using a Qubit® 2.0 Fluorometer (Life Technologies) and integrity by migrating about 100 ng of GelRed™-stained DNA on an agarose 1.0% gel. DNA used for genome sequencing was subjected to quality/purity control using the UV/Vis measurements of the Nanodrop™ 2000 (Thermo Fisher Scientific™), quantified using the Qubit DNA BR Assay kit (cat # Q32850, Thermo Fisher Scientific), and its integrity assessed with the Femto Pulse Genomic DNA 165 kb kit (Agilent). RNA was extracted with mirVana™ miRNA Isolation Kit (cat # AM1560, Invitrogen™). RNA was quantified by Qubit RNA BR Assay kit (Thermo Fisher Scientific) and its integrity was estimated by using RNA 6000 Nano Bioanalyzer 2100 Assay (Agilent).

### Library preparation and sequencing

The *S. scombrus* genome assembly was produced using a combination of genomic and transcriptome long and short reads. Whole genome long-read sequencing libraries were prepared using the SQK-LSK110 1D sequencing kit from Oxford Nanopore Technologies™ (ONT). The sequencing run was performed on a PromethIon™ 24 instrument (ONT) using a flow cell R9.4.1 FLO-PRO002 (ONT). Transcriptome long read sequencing libraries were prepared using the cDNA-PCR Sequencing Kit SQK-PCS111 (ONT), following the manufacturer's instructions. Sequencing runs were performed on GridION™ Mk1 (ONT) using a Flowcell R9.4.1 FLO-MIN106D (ONT). The quality parameters of the sequencing runs, for both long reads and cDNA, were monitored in real time using the MinKNOW™ platform (version 21.10.8 for long reads and version 22.05.7 for cDNA), and the base calling was performed using Guppy (ONT, version 6.2.7 and version 6.1.5 respectively, https://community.nanoporetech.com/downloads) “super-accuracy” mode (“dna_r9.4.1_450bps_sup.cfg” config file). The free alternative open-source high performance base calling software Dorado ( https://github.com/nanoporetech/dorado) can also be used to base call the reads. Whole genome short-read sequencing Illumina platform compatible libraries were prepared using the PCR-free protocol from KAPA HyperPrep kit (Roche). Following end-repair and adenylation, Illumina platform-compatible adapters containing unique dual indexes and unique molecular identifiers (Integrated DNA Technologies) were ligated. The transcriptome short-read libraries were prepared with KAPA Stranded mRNA-Seq Illumina Platforms Kit (Roche) following the manufacturer´s recommendations starting with 500 ng of total RNA as the input material. The libraries were quality controlled on an Agilent 2100 Bioanalyzer with the DNA 7500 assay (Agilent) to assess size and quantified using the Kapa Library Quantification Kit for Illumina platforms (Roche). Whole genome and transcriptome short-read libraries were sequenced on an Illumina NovaSeq™ 6000 with a read length of 2x150bp, following the manufacturer’s protocol for dual indexing. Image analysis, base calling and quality scoring of the run were processed using the manufacturer’s software Real Time Analysis (RTA 3.4.4). Generated raw long and short genome and transcriptome paired-end reads are available at the European Nucleotide Archive (ENA; accession number: PRJEB70238).

Restriction-site-associated DNA (RAD) libraries were prepared following the methods of Etter *et al.* (2011). Namely, between 50 and 750 ng of starting DNA (depending on availability and integrity) was digested with the SdaI (SbfI) restriction enzyme (Thermo Fisher Scientific, cat # ER1191) in 20 µl reactions including 17 µl DNA, 1 µl Enzyme SdaI (SbfI) and 2 µl of 10X buffer. The mix was incubated 2 hours at 37°C, followed 20 minutes at 80°C to inactivate the enzyme. After digestion, Illumina-compatible P1 adapter, which contains 5bp sample-specific barcodes for samples demultiplexing, was ligated. The reaction was done in 30 µl using T4 DNA Ligase (Thermo Fisher Scientific, cat# EL0013), with 20 µl of digested DNA, 0.5 μl NEB Buffer 2 (10X, cat# B7002S)), 0.3 μl rATP (100mM, Thermo Scientific™ cat# R0441), 1 μl Ligase (30U/ μl, Thermo Scientific™ cat# EL0013), 2 μl adapter P1 (100nM) and water. The mix was incubated 16 hours at 22°C, followed 10 minutes at 65°C. Pools of 32 individuals were created and purified using Genejet PCR Purification kit (Thermo Fisher Scientific, cat# K0701). Each pool was sheared using the Covaris® M220 Focused-ultrasonicator™ Instrument (Life Technologies) with a default method “DNA_0500_bp_130_μl_Snap_Cap_microTUBE” (PowerPeak 50.0; Duty Factor 10.0; Cycles/Burst 200; Time 00h:01m:00s) and size selected to 300-500 bp by cutting agarose gel migrated DNA. The bands were extracted using the Genejet Purification kit (Thermo Fisher Scientific, cat# K0701) and eluting in 25.5 μl of Milli-Q water. Purified products were then End repaired (Thermo Fisher Scientific, cat# K0771) incubating at 20°C for 20 minutes the following mix: 25.5 μl of pool, 3 μl End Repair Reaction Mix (10X) and 1.5 μl End Repair Enzyme. Following, 3’-dA Overhang Addition was performed by incubating the end-repaired products at 37°C for 30 minutes (30 μl) with 5 μl of NEB Buffer 2 (10X), 1 μl of dATP (10mM, Thermo Fisher Scientific, cat# R0141) and 3 Klenow Fragment, exo– (5 U/μL, Thermo Fisher Scientific, cat# EP0422). Before amplification, Illumina different P2 adaptors (10 μM) were ligated to each Pool in the same conditions as the P1 ligation step.

Finally, each library was amplified in triplicates in volume of 25μl, using 5 μl of sample, 12.5 μl High Fidelity Phusion Master mix (Thermo Fisher Scientific, cat# F531S) and 1 μl of PCR primers (10 μM, PCR-For 5´-AATGATACGGCGACCACCGA-3´ and PCR-Rev 5´-CAAGCAGAAGACGGCATACGA-3´). The PCR profile was 98°C- 3minutes and 14 cycles of 98°C-15s; 65°C-30s;72°C-30s. Each replicate was mixed and purified in ratio 1:1.8 (sample/beads) with Axyprep MAG PCR Clean (Axygen®, cat # Mag-PCR-CL-50). Batches of three pools were paired end sequenced (100 bp) on an Illumina HiSeq2000.

### Genome assembly and annotation

The genome assembly and annotation workflows are schematized in Supplementary Figures S1 and S2 in *Extended data* ( Manuzzi *et al.*, 2024). Prior to assembly, the DNA Illumina short reads were trimmed for adaptors using TrimGalore v0.6.7 ( https://github.com/FelixKrueger/TrimGalore) and the ONT long read genomic data was filtered to remove short and low-quality reads with Filtlong2 v0.2.1 ( https://github.com/rrwick/Filtlong) with parameters: --minlen 700 --min_mean_q 80. Resulting ONT reads were assembled with Flye v2.9 ( Kolmogorov *et al.*, 2019) using the ‘nano-raw’ mode and a minimum overlap of 1000. Illumina short reads and ONT were mapped against the generated assembly using BWA-mem v2.2.1 ( Li & Durbin, 2009) and MINIMAP2 v2.24 ( Li, 2018) respectively to assess contigs continuity and completeness as well as to identify possible base accuracy errors. The assembly was polished with HyPo v1.0.3 ( Kundu *et al.*, 2019) to improve base accuracy using both Illumina and ONT data. Finally, the polished assembly was purged with purge_dups v1.2.6 ( Guan *et al.*, 2020) to remove alternate haplotypes and other artificially duplicated repetitive regions. The consensus quality (QV) of the final assembly was estimated by Merqury v1.3 ( Rhie *et al.*, 2020) and the gene completeness by BUSCO v5.4.0 ( Manni *et al.*, 2021) using the odb10_actinopterygii database. QV represents the probability of error on a logarithmic scale for the consensus of the called bases. The higher the QV the higher the consensus, e.g. Q40 corresponds to a 99.99% accuracy ( Rhie *et al.*, 2020). The histogram of the k-mer counting distribution was plotted in GenomeScope 2.0 v2.0 ( Ranallo-Benavidez *et al.*, 2020).

The annotation of the assembly was obtained by combining transcript alignments, protein alignments and *ab initio* gene predictions. Repeats present in the genome assembly were annotated with RepeatMasker v4-1-2 ( http://www.repeatmasker.org) using the custom repeat library available for *Danio rerio*. Moreover, a new repeat library specific for our assembly was made with RepeatModeler v1.0.11. After excluding those repeats that were part of repetitive protein families (performing a BLASTref search against Uniprot) from the resulting library, RepeatMasker was run again with this new library to annotate the specific repeats. The transcriptome short Illumina and long ONT reads were aligned to the genome using, respectively, STAR v-2.7.10a ( Dobin *et al.*, 2013) and MINIMAP2 v2.24 ( Li, 2018) with the splice option after which high-quality junctions to be used during the annotation process were obtained by running Portcullis v1.2.4 ( Mapleson *et al.*, 2018). Transcript models were subsequently generated using Stringie v2.2.1 ( Pertea *et al.*, 2015) on each BAM file and then all the models produced were combined using TACO v0.7.3 ( Niknafs *et al.*, 2017). Finally, PASA assemblies were produced with PASA v2.5.2 ( Haas *et al.*, 2008), and the *TransDecoder* program, which is part of the PASA package, was run on the PASA assemblies to detect coding regions in the transcripts. The complete proteomes of *Carassius auratus, Cynoglossus semilaevis, Danio rerio, Oryzias latipes, Parambassis ranga, Sparus aurata* and *Scopthalmus maximus* were downloaded from Uniprot in March 2022 and aligned to the genome using Miniprot 0.6 ( Li, 2023). *Ab initio* gene predictions were performed on the repeat-masked assembly with three different programs: GeneIDv1.4 ( Alioto *et al.*, 2018), Augustus v3.5.0 ( Stanke *et al.*, 2006) and Genemark-ET v4.71 ( Lomsadze *et al.*, 2014) with and without incorporating evidence from the RNAseq data. The gene predictors were run with trained parameters for human, except Genemark, which runs in a self-trained mode. Finally, all the data were combined into consensus CDS models using EvidenceModeler-1.1.1 (EVM; Haas *et al.* (2008)). Additionally, UTRs and alternative splicing forms were annotated via two rounds of PASA annotation updates. Functional annotation was performed on the annotated proteins with Blast2go v1.3.3 ( Conesa *et al.*, 2005) after a Blastp (Diamond Blastp v2.0.15, Buchfink *et al.*, 2021) search against the nr database (last accessed March 2023) and an Interproscan v5.55_88.0 ( Jones *et al.*, 2014) run to detect protein domains on the annotated proteins. The annotation of non-coding RNAs was obtained by running cmsearch v1.1.4 ( Cui *et al.*, 2016), that is part of the Infernal ( Nawrocki & Eddy, 2013) package, against the RFAM database of RNA families v12.0 ( Nawrocki & Eddy, 2013). Additionally, tRNAscan-SE v2.0.11 ( Chan & Lowe, 2019) was run in order to detect the transfer RNA genes present in the masked genome assembly. Identification of long non-coding RNAs was done by first filtering the set of PASA-assemblies that had not been included in the annotation of protein-coding genes to retain those longer than 200bp and not covered more than 80% by a small ncRNA. The resulting transcripts were clustered into genes using shared splice sites or significant sequence overlap as criteria for designation as the same gene. BlobToolKit INSDC pipeline ( Challis *et al.*, 2020) was run on the assembled genome using the NCBI nt database (updated on May 2023) and the following BUSCO odb10 databases: Actinopterygii, Vertebrata, Metazoa, Eukarya, Fungi and Bacteria. The genome assembly and annotation are available in the ENA under the accession number “GCA_963921475.1”. Additionally, all the files resulting from the annotation process and a genome browser can be found in https://denovo.cnag.cat/fScoSco.

### RAD-loci assembly and genotype table generation

Generated RAD-tags were analysed using *Stacks* version 2.4 ( Catchen *et al.*, 2013). Quality filtering and demultiplexing were performed using the module *process_radtags* with default parameters, removing reads with adaptor sequences and truncating to 90 bp maximum length to further exclude low quality bases. Only reads whose forward and reverse pair passed quality filtering were kept and the module *clone_filter* was applied to remove PCR duplicates. Clean reads were aligned to the newly assembled Atlantic mackerel genome (see above) using BWA-mem 0.7.17 ( Li & Durbin, 2009) with default parameters. Mapped reads were then deduplicated using Picard 2.0.1 MarkDuplicates ( http://broadinstitute.github.io/picard/). Samples exceeding a 25% duplication level were excluded from downstream analysis. The module *gstacks* was used to assemble paired-end reads into contigs, merging them to the single-end loci and identifying and genotyping SNPs. The module *populations* was used to select the RAD-loci found in at least 90% of the individuals, sorted by reference order to ensure output is sorted according to the genome coordinates of the reference genome and possibly overlapping sites are not maintained (variant sites are printed only once for every genomic position). VCFtools v.0.1.13 ( Danecek *et al.*, 2011) was used to select samples and SNPs with a minimum of 0.75 and 0.95 genotyping rate respectively and SNPs with minimum allele frequency (MAF) larger than 0.05. Kin pairs were detected through a genetic relatedness matrix estimated using VCFtools based on the values of relatedness calculated between all pairs of individuals using the Akj model ( Yang *et al.*, 2010). Seven pairs involving overall 14 individuals with relatedness coefficients between 0.66 to 1 were found, of which individuals with the highest level of missing data from each pair were removed for downstream analysis. An additional pair of samples with 0.16 relatedness coefficient was also removed for being divergent with respect to the remaining pairwise comparisons (all < 0.08). After removal of these eight individuals, the module *populations* as well as downstream filtering steps were run again with the same parameters as above on the overall dataset as well as for each subset of individuals to be analysed.

### Population structure analyses

Spatial structure inferences and neutrality tests were performed for the overall dataset (East and West Atlantic, and Mediterranean Sea; N=515, after filtering) as well as for the different spatial subsets (Atlantic only N=417, NWA N=150, NEA N=273). In addition, two further subsets were created including only reference samples, namely larvae and spawning adults (LSA), to investigate connectivity between spawning component (NEA-LSA N=170, NWA-LSA N=59; Supplementary Table S1 in *Extended data* ( Manuzzi *et al.*, 2024). The genetic ancestry of each individual was estimated on the overall dataset filtered for physical linkage (1 SNP each 1kb window) using the model-based clustering method implemented in ADMIXTURE 1.3.0 ( Alexander *et al.*, 2009) assuming from 2 to 18 ancestral populations (K) and setting 1,000 bootstrap runs. The linkage filter was performed by using a customized R script (“ld_pruning.r”; Manuzzi *et al.* (2024)) as suggested by ADMIXTURE manual since the software does not account for LD and thus it may produces false positives. The value of K with the lowest associated error value was identified using ADMIXTURE’s cross-validation (CV) criterion. Principal Component Analyses (PCAs) were performed using the package *adegenet* 2.1.10 ( Jombart & Ahmed, 2011) in R version 4.1.2 ( Team, 2022) and visualized using *ggplot2* 3.5.1 ( Wickham, 2016) and *factoextra* 1.0.7 ( Kassambara & Mundt, 2020) packages. Estimates of pairwise F _ST_ between groups were performed using the R package StAMPP 1.6.3 ( Pembleton *et al.*, 2013) after removing populations with reduced sample size (<7, GREEN N=2) and their significance was assessed with 1,000 permutations over loci. For all FST comparisons the p-values were adjusted using a False Discovery Rate (FDR) method. Loci potentially under selection were screened for the different spatial subsets (ATL, NEA, NWA) and for the subsets including only reference samples (NEA-LSA, NWA-LSA) (Supplementary Table S2; Manuzzi *et al.* (2024)) using the multivariate approach implemented in the R package *pcadapt* 4.3.3 ( Luu *et al.*, 2017) setting alpha value to 0.05 as cut-off.

## Results

### Genome assembly and annotation

**Figure 1.**
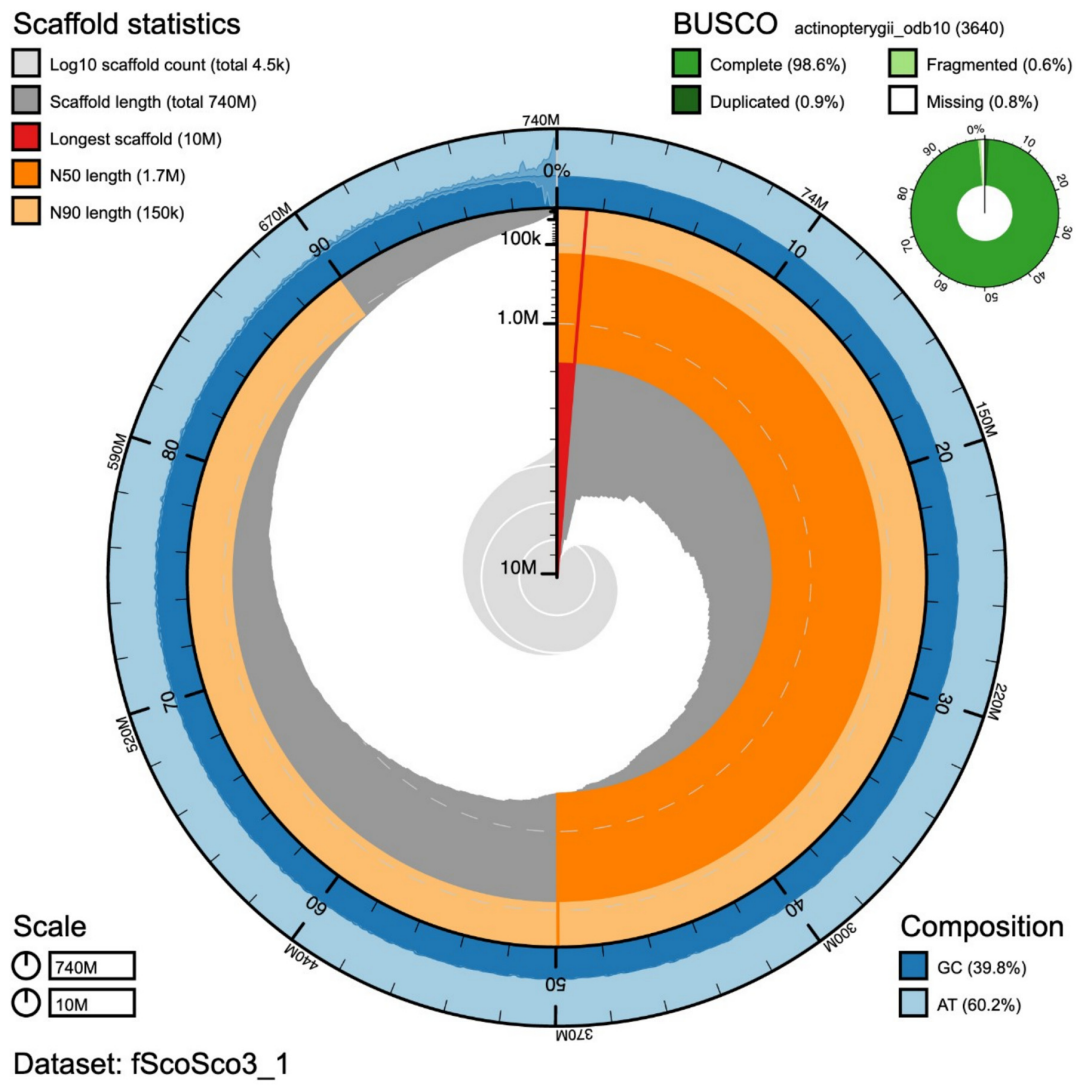
Snailplot summary of the assembly metrics for the *Scomber scombrus* genome. The main plot is divided into 1,000 size-ordered bins around the circumference with each bin representing 0.1% of the 741,290,950 bp assembly. The distribution of scaffold lengths is shown in dark grey with the plot radius scaled to the longest scaffold present in the assembly (10,208,092 bp, shown in red). Orange and pale-orange arcs show the N50 and N90 scaffold lengths (1,748,220 and 152,403 bp), respectively. The pale grey spiral shows the cumulative scaffold count on a log scale with white scale lines showing successive orders of magnitude. The blue and pale-blue area around the outside of the plot shows the distribution of GC, AT and N percentages in the same bins as the inner plot. A summary of complete, fragmented, duplicated and missing BUSCO genes in the actinopterygii_odb10 set is shown in the top right.

The Atlantic mackerel estimated genome size was of 769Mb with 0.68% heterozygosity. The raw assembly we obtained comprises a total of 765Mb in 9,496 contigs, while the final assembly (after polishing and purging), 741Mb in 4,482 contigs. The N50, namely the length of the shortest contig at 50% of the total assembly length, of the final assembly is 1.7 Mb and the L50, the smallest number of contigs required to make up half of genome size, is 110 with the longest contig measuring 10,208,092 bp. The consensus quality (QV) of the final assembly was estimated at 41.4 with 98.6% gene completeness reported. A statistical representation of the genome assembly is shown in Figure 1 in the form of a snail plot. In total, we annotated 26,428 protein-coding genes that produce 38,000 transcripts (1.44 transcripts per gene) and encode for 35,860 unique protein products. We were able to assign functional labels to 94.2% of the annotated proteins. The annotated transcripts contain 10.88 exons on average, with 93% of them being multi-exonic ( Table 2). In addition, 7,871 non-coding transcripts were annotated, of which 6,063 and 1,808 are long and short non-coding RNA genes, respectively. Summarizing assembly and annotation statistics are reported in Table 1 and Table 2, respectively, and the results on the genome coverage distribution are reported in Supplementary Figure S3 ( Manuzzi *et al.*, 2024) and show two 20-mer peaks of coverage at 33 (1n, heterozygous) and at 66 (2n, homozygous).

**Figure 2.**
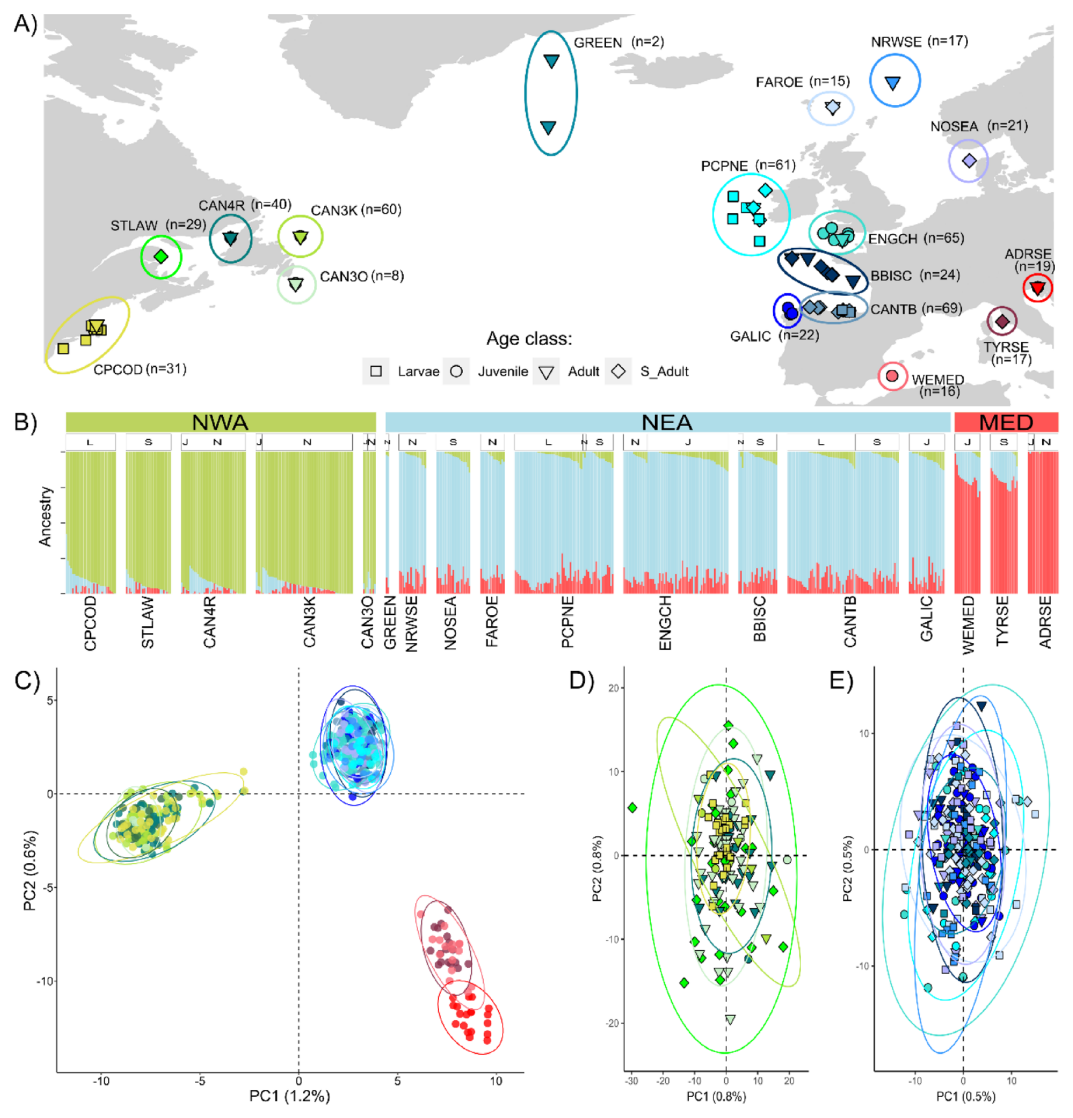
Population structure analyses. ( **A**) Map of sampling collections included in the study. Each colour represents a sampling area. Names and sample sizes of each group are reported in the map. Different shapes are used to identify different life stages (larvae, juvenile, adult, and spawning adult). ( **B**) Admixture results for best K=3. Levels of admixture are shown on the y-axis and are ordered by Q-values within each group and for each life stage. Life stages are coded on top of the admixture plot as: L=larvae, J=juvenile, N=adult not-spawning, S=spawning adult. In green, the western Atlantic ancestry component, in sky-blue, the eastern Atlantic, and in red the Mediterranean one as estimated by ADMIXTURE. ( **C**) Principal component analyses (PCA) performed on individuals of the Atlantic mackerel based on the genomic dataset of 24,271 SNPs and 515 individuals in the overall dataset and for the different age classes within the NWA ( **D**; 150 individuals, 39,541 SNPs) and NEA ( **E**; 273 individuals, 39,528 SNPs).

**Table 1.** Genome assembly statistics. Results obtained after polishing steps performed using HyPo4 with both Illumina and ONT data, and purging using purge_dups5 to remove alternate haplotype and duplicated repetitive regions. The final assembly comprised of 741Mb and 4,483 contigs scaffolds.

Assembly	Flye + hypo + purged
N50	1,748,220 bp
L50	110
Total sequences	4,482
Total length	741,290,950 bp
BUSCO* complete	98.6%
BUSCO* duplicated	0.9%
QV	41.4058
Kmer completeness	88.873

**Table 2.** Genome annotation statistics. The gene annotation of the mackerel genome assembly was obtained by combining transcript alignments, protein alignments and ab initio gene predictions.

	Annotation
Number of protein-coding genes	26,428
Median gene length (bp)	7,720
Number of transcripts	38,000
Number of exons	278,035
Number of coding exons	264,221
Median UTR length (bp)	1,142
Median intron length (bp)	431
Exons/transcript	10.88
Transcripts/gene	1.44
Multi-exonic transcripts	93%
Gene density (gene/Mb)	35.65

### RAD-loci assembly and genotype table generation

After sequencing, we obtained an average of 6,108,766 raw reads per sample (minimum=411,982; maximum=26,351,634). Following mapping, 60 specimens were excluded from downstream analyses because of > 50% duplicated reads, < 20% of properly paired reads and/or median insert size <10bp. For the remaining individuals, an average of 99.14% of the filtered reads aligned to the reference genome. After SNPs calling, eight additional samples were removed due to high relatedness values (Methods) and further 23 due to missing data >25%. Finally, seven genotype tables were generated with a variable number of samples (59–515) and SNP markers (10,888–39,541) depending on the purpose of the analysis (Supplementary Table S2; Manuzzi *et al.* (2024)) and with an average SNP coverage, calculated over all samples for each dataset, of 16X–23X.

### Population structure analyses

Based on the results of the ADMXITURE test, K=2–3 were equally likely numbers of genetic clusters within our collection, corresponding to the lowest cross-validation errors (CV; Supplementary Figure S4.A; Manuzzi *et al.* (2024)). From the ADMIXTURE plot, the eastern Atlantic appears to more likely have shared ancestral origin with the Mediterranean samples, with the greatest difference existing between the Mediterranean and the western Atlantic collections (K=2, Supplementary Figure S4.B; Manuzzi *et al.* (2024)). Increasing the number of ancestral populations (K=3, Figure 2.B) showed three clusters with distinct genetic ancestry, corresponding to the geographical separations, namely NWA (green), NEA (sky-blue) and MED (red). No differentiation was detected within each cluster, and the three lineages coinciding with the geographical division described the full extent of the structure with individual samples showing high ancestry proportion (Q) within each respective cluster. Individual ancestry proportions do not appear to be correlated with the latitudinal gradients nor with the different age classes (L=larvae, J=juveniles, N=non-reproductive adults and S=reproductive adults) visible in the upper bar of the ADMXITURE plot. This geographical separation is confirmed by the results of the PCA ( Figure 2.C), where a clear differentiation between the same three genetically separated groups is apparent, with the first and second components of the PCA explaining 1.2% and 0.6% of the variance respectively. All pairwise comparisons between the three groups resulted in high F _ST_ values (p-values=0.000), with the highest value reported for MED vs NWA (F _ST_=0.035), as expected given their geographical distance, and the lowest between NEA and NWA (F _ST_=0.014), closely followed by the NEA vs MED comparison (F _ST_=0.019). However, beyond the NWA-NEA Atlantic divide, the PCAs further confirm the lack of further substructure within the respective Atlantic basins (NWA Figure 2.D; NEA Figure 2.E), where the variance is minimal and explained in equal proportions by the first two axes in both within-basin analyses (NWA, PC1=PC2=0.8%; NEA, PC1=PC2=0.5%). Pairwise F _ST_ values ranged from 0.000 to 0.001 within the NEA even for the more spatially distant areas, and from 0.000 to 0.003 within NWA (Supplementary Tables S3–S4; Manuzzi *et al.* (2024)). In the Mediterranean Sea, instead, local structure is discernible in both the ADMIXTURE and the PCA analyses where the Adriatic Sea appears genetically distinct from the remaining western Mediterranean collections and is composed of genotypes of pure Mediterranean ancestry. This differentiation within the Mediterranean Sea is also seen in the F _ST_ pairwise comparisons, with significant (p-values=0.000) F _ST_ values of 0.003 and 0.004 for the Adriatic Sea versus the Tyrrhenian Sea and Western Mediterranean Sea respectively. Finally, all population structure analyses clearly cluster the Greenlandic samples with the NEA ( Figure 2.B-C-D) confirming the eastern Atlantic origin of the samples currently harvested in the relatively recent northward migration ground. Although outlier markers were identified for the Atlantic (275 SNPs), Northwest Atlantic (65) and Northeast Atlantic (38), lack of sub structuring was maintained when analysing only these markers (Supplementary Figure S5; Manuzzi *et al.* (2024)). Additionally, even when including only reference samples, no evidence of a genetically based differentiation was identified neither by neutral nor putative outlier markers (Supplementary Figure S6; Manuzzi *et al.* (2024)), with significant (p-value=0.000) F _ST_ values of 0.0004 and 0.001 between larvae and spawning adults within the NEA and NWA, respectively.

## Discussion

Here, we present a reference genome for the Atlantic mackerel, as well as the most comprehensive SNP dataset and most broadly distributed samples used to resolve the questions left unanswered regarding the population structure and connectivity of this species.

### Three main genetically isolated populations in Atlantic mackerel

Our results confirmed the presence of isolated populations between the two sides of the North Atlantic Ocean, as well as between the Atlantic and the Mediterranean Sea with no or reduced genetic exchange between the three distinct groups, which settles previous studies ( Bourret *et al.*, 2023; Gíslason *et al.*, 2020; Nesbø *et al.*, 2000; Rodriguez-Ezpeleta *et al.*, 2016; Zardoya *et al.*, 2004). Moreover, we confirm that mackerel found in Greenland are part of the NEA population ( Bourret *et al.*, 2023; Gíslason *et al.*, 2020). Despite inter-population F _ST_ values being low (F _ST_=0.014–0.035), these are in the range of what is usually observed in marine fish species with large population sizes since F _ST_ estimates are affected by both effective population sizes and migration rates, which may derive into insufficient statistical power provided by neutral markers to reject the panmictic hypothesis ( Waples, 1998). Furthermore, low F _ST_ values do not always indicate high migration rates between populations and may also reflect the presence of a large metapopulation with high effective population size and low migration rates ( Gagnaire *et al.*, 2015); further studies are required to decipher these dynamics.

Thus, despite the use of a large dataset with tens of thousands of SNP markers and a reference genome, no further signs of population structure were detected within the NEA or the NWA. Only in the Mediterranean Sea, differentiation between the Adriatic Sea and the western Mediterranean was found, confirming previous studies ( Rodriguez-Ezpeleta *et al.*, 2016). This supports the hypothesis of high mixing and large effective population size.

### A single population within the Northeast Atlantic mackerel: implications for management

The lack of fine-scale genetic structure allowed us to reject the hypothesis of strict homing behaviour genetically isolating the NEA mackerel spawning areas. Under this hypothetical scenario, genetic differentiation would be maintained despite the demographic connectivity supported by the migratory behaviour as movement would not derive into gene flow ( Dittman & Quinn, 1996). Unlike the NWA ( Bourret *et al.*, 2023), none of the analyses conducted to date trying to disentangle the population structure of NEA mackerel have been based on thousands of genome-wide markers, nor have been successful in providing compelling evidence for the presence or absence of genetically isolated spawning components ( Nesbø *et al.*, 2000). Our large dataset, both in number of individuals and markers, settles doubts on the population structure of Atlantic mackerel in the NEA, and reject the hypothesis of homing behaviour. Indeed, our analyses find no evidence of genetically isolated spawning components even when using only larvae and adults, which are supposed to represent the spawning population at one location, or outlier markers, that is, those potentially under selection. These results do not support a mixed stock fishery scenario as that found in other species (e.g. Atlantic bluefin tuna; Rodríguez-Ezpeleta *et al.* (2019)), which would require the need for developing a genetic stock identification method. Indeed, our study supports the use of a single stock for NEA mackerel assessment, which is the approach currently used.

Yet, it should be noted that our work does not include samples from southern Europe (e.g. south of Portugal) which would allow to further investigate the connectivity between southern NEA and and the North Atlantic African coast and for which no conclusion can be drawn. Additionally, we cannot exclude the possibility that our dataset fails to include a reduced number of genetic markers that might be involved in differentiating contingents, that have not been targeted by our RAD sequencing approach. This has been seen, for example, in the Atlantic herring ( Han *et al.*, 2020), for which early genetic studies were unable to disentangle population differentiation, nor to discern adaptive markers from the neutral background, due to the high levels of gene flow and large effective population sizes. However, once whole genome sequencing (WGS) data were used, differences associated with a reduced number of genomic regions were detected in both Atlantic herring and Atlantic horse mackerel. For the Atlantic herring ( Han *et al.*, 2020), fewer regions were found to be associated with an adaptation to seasonal reproduction allowing the differentiation of reproductive components despite the overall weak genomic differentiation, whereas in the Atlantic horse mackerel ( Fuentes-Pardo *et al.*, 2023) fewer regions under selection revealed geographic differences associated with environmental variables.

### Forecasting changes

The highly migratory behaviour, coupled with the wide species distribution, entails that the Atlantic mackerel can occupy several different environments during its life, suggesting that the species may be adapted to a wide range of temperatures. The lack of differentiation within the NEA supports the hypothesis of a high dispersal capacity of the species, further confirmed by the Greenland samples that appear to have originated from a north-westward migration of NEA mackerel ( Jansen *et al.*, 2016). In addition, the species shows high plasticity as it can tolerate up to 5–15°C ( Olafsdottir *et al.*, 2019) despite its optimal temperature range (9–13°C), which is possibly linked to the high genomic heterozygosity detected at the species level. Here, our findings do not show signs of selection associated to this shift, and no putative outlier markers were detected. These results, together with observed northward shift of distribution ( Olafsdottir *et al.*, 2019) and spawning area ( Chust *et al.*, 2023; dos Santos Schmidt *et al.*, 2023) linked to water temperature variations suggest a future displacement of NEA mackerel under climate scenarios of a progressively warmer Atlantic with forecasted spawning distribution shifts both westwards and northwards ( Bruge *et al.*, 2016). Despite the low sample size, the Greenlandic samples in our collection are clearly part of the NEA population, and no connectivity with NWA was detected here. However, if the species' range keeps expanding it cannot be excluded that a bridge in connectivity at northern latitudes may happen, since mackerel from the NWA population have been found as far north as Labrador ( Parsons, 1970). Indeed, warmer climate creating thermal niches suitable for expansion could bring isolated populations closer thus reducing the existing barrier to gene flow and allowing for secondary contact to occur. This is usually referred to as an interspecific contact, however it could occur between naturally isolated populations of the same species (intraspecific). Reported cases of recent interspecific secondary contact are so far limited (e.g. hybridization by human-driven translocation of *Ciona robusta* to its congeners’ distribution areas, Le Moan *et al.* (2021)), especially as a result of recent climate change (e.g. hybridization due to recent distribution shift causing contact during spawning events *Argyrosomus coronus*, Potts *et al.* (2014)) and none is reported for intraspecific contact due to the disappearance of a previously existing barrier. Indeed, population shifts and the creation of contact zones between populations and/or species usually occur over geological timescales, but this could change and accelerate with the current acceleration and magnitude of ocean warming ( Pinsky *et al.*, 2020).

### Future Atlantic mackerel genomic studies

The availability of a species reference genome could help to further investigate the presence of distinct evolutionary significant units for management linked to adaptive potential, thereby identifying associated gene families, and to assess the demographic history of identified areas of differentiation (e.g. the one that caused the Adriatic split). In recent years, high-throughput sequencing has made it more cost-effective to produce reference genomes even for non-model organisms. Here, we have used three generally accepted metrics to assess genome quality: contiguity, completeness, and correctness ( Lewin *et al.*, 2018; Wang & Wang, 2023) and found that the Atlantic mackerel genome produced here follows high quality standards. This resource will be highly valuable for future studies on this species, such as those based on whole genome resequencing data, which may reveal further complexity not detected when using reduced representation datasets. The evidence for the northward expansion of the distribution range of the species, and potential secondary contact between NEA and NWA, highlight the need for future genetic monitoring of this species, especially on newly occupied areas, to detect and estimate potential population mixing ( Schwartz *et al.*, 2007).

## Ethics and consent

Ethical approval and consent were not required.

## Data Availability

The data used in this study are publicly available at: Sequence Read Archive (SRA, NCBI): Demultiplexed RAD sequences of Atlantic mackerel included in this study. Accession number: PRJNA1081273; https://www.ncbi.nlm.nih.gov/bioproject/1081273 ( Manuzzi *et al.*, 2024). European Nucleotide Archive (ENA): Long and short genome and transcriptome paired-end reads used for the genome assembly and annotation. Accession number: PRJEB70238; https://www.ebi.ac.uk/ena/browser/view/PRJEB70238 ( Manuzzi *et al.*, 2024). Zenodo: Data from: Atlantic mackerel population structure does not support genetically distinct spawning components. https://zenodo.org/doi/10.5281/zenodo.10684820 ( Manuzzi *et al.*, 2024). This project contains the following supplementary data: “Table S1.xlsx” including all metadata for the samples included in the population structure and adaptation analyses performed in the manuscript. The table contains 20 columns: AnalisisID, Barcode, Pool, Area, Region, Pop, Age, Size(mm), Sex, Maturity stage, Latitude, Longitude, Collection date, and each dataset tested reporting the list of samples included. “Extended_data.docx” containing Supplementary Tables and Figures. In term of tables, it includes: (1) Table S2: Table reporting the samples’ numbers, SNPs’ number and filtering steps of each genotype tables (Dataset) used. (2) Table S3. . Pairwise FST (lower diagonal) and respective p-values (upper diagonal) between the different populations within the NEA. (3) Table S4. Pairwise FST (lower diagonal) and respective p-values (upper diagonal) between the different populations within the NWA. Additionally, it includes (5) Figure S1. Workflow of the genome assembly process, (6) Figure S2. Workflow of the genome annotation process, (7) Figure S3. Genoscope transformed linear plot, (8) Figure S4. Cross-validation for ADMIXTURE test, (9) Figure S5. PCA plots of the outlier SNPs detected by pcadapt and (10) Figure S6. PCA plots of neutral and outlier SNPs detected using reference samples from NEA and NWA Scripts used to perform the analyses described in this manuscript. Data are available under the terms of the Creative Commons Attribution 4.0 International license (CC-BY 4.0).
