# Supplementary material for: Atlantic mackerel population structure does not support genetically distinct spawning components
Source: Open Res Eur. 2025 Jan 20;4:82. Originally published 2024 Apr 24. [Version 2] doi: 10.12688/openreseurope.17365.2 (PMC11544206; doi:10.12688/openreseurope.17365.2)
Supplement: Peer review history

# Reviewer report: Reviewer response for version 2

**DOI:** 10.21956/openreseurope.20953.r50707

**Published:** 2025-02-20

**Authors:** John S Hargrove

**Affiliations:** 1 Pacific States Marine Fisheries Commission, Trout Rd, Eagle, USA

In their article titled "Atlantic mackerel population structure does not support genetically distinct spawning components”, Manuzzi et al. generated an annotated genome for the Atlantic mackerel, collected reduced representation sequence data consisting of tens of thousands of SNPs, and used these data to test for the presence of genetic structuring within the spawning components of the northeast Atlantic Ocean and northwest Atlantic Ocean. The authors provide excellent justification for the study, noting that Atlantic mackerel are of significant economic importance, that climate change-related range expansion has led to management complications, and that ambiguity remains regarding the genetic composition of individual spawning components despite previous research studies. The authors note the utility of a reference genome as this resource facilitates novel forms of downstream analysis (e.g. detection of genomic regions under selection) which may further advance conservation and management efforts.

 Overall, I was very impressed with the breadth and quality of this study. The construction of a reference genome is a major accomplishment in and of itself, and the methodologies used to sequence, assemble, and annotate the genome were well-established and appropriate. Access to a reference genome minimized potential complications associated with *de novo* locus identification, thus bolstering the quality of the data used for population structure analysis. I found the statistical approaches to assess population structure and the genetic relationships between spawning components were appropriate and correctly applied. Lastly, I thought the authors did an excellent job interpreting the results and placing them in the context of existing knowledge. I applaud the researchers on their work!

Is the study design appropriate and does the work have academic merit?

Yes

Is the work clearly and accurately presented and does it cite the current literature?

Yes

If applicable, is the statistical analysis and its interpretation appropriate?

Yes

Are all the source data underlying the results available to ensure full reproducibility?

Yes

Are the conclusions drawn adequately supported by the results?

Yes

Are sufficient details of methods and analysis provided to allow replication by others?

Yes

Reviewer Expertise:

My research focuses broadly on population genetics with a particular emphasis on genetic stock structure, mating systems, and inter/intraspecific hybridization.

I confirm that I have read this submission and believe that I have an appropriate level of expertise to confirm that it is of an acceptable scientific standard.

# Reviewer report: Reviewer response for version 2

**DOI:** 10.21956/openreseurope.20953.r50706

**Published:** 2025-02-11

**Authors:** Gaetano Catanese

**Affiliations:** 1 IRFAP LIMIA (Laboratorio de Investigaciones Marinas y Acuicultura) – Govern de les Illes Balears, Balearic Islands, Spain

In this study, the authors sequenced and annotated the genome of *Scomber scombrus* to investigate population structure and potential genetic adaptations. Using RAD-seq data from over 500 samples, the analysis of SNPs confirmed the existence of three genetically distinct units (Northwest Atlantic, Northeast Atlantic, and Mediterranean) but found no evidence of separate spawning components within the Atlantic. These findings could clarify previous uncertainties, rejecting homing behavior and the need to redefine management boundaries for the species.

 First of all, I would like to commend the authors for their outstanding work in developing this manuscript. The study is well-structured, presenting a clear and logical progression from the introduction to the conclusions. The introduction effectively contextualizes the research question, providing a solid theoretical background and a well-articulated justification for the study. The discussion is equally well-developed, offering a critical interpretation of the findings in light of existing literature, while also highlighting their significance and potential implications.

 A particularly strong aspect of this work is the robustness of the methodology and the comprehensiveness of the analyses. The results are supported by rigorous statistical and analytical approaches, which enhance the reliability and credibility of the conclusions drawn. The authors have demonstrated great attention to detail in ensuring that the findings are presented clearly and transparently.

 Additionally, I would like to acknowledge the thorough and detailed review process carried out by the other reviewers. Their insightful comments and suggestions have contributed significantly to refining the manuscript. The authors have responded thoughtfully to all critiques, implementing the requested revisions in a precise and constructive manner. Their efforts in addressing ambiguities and improving clarity are evident, further strengthening the overall quality of the work.

 However, I would like to offer a comment and recommendation to enhance the manuscript, specifically regarding the genetic differentiation of the samples.

 In response to another reviewer's request, the authors included pairwise FST comparisons between Mediterranean and Atlantic groups in the Results section. However, I believe it would be valuable to provide a more comprehensive FST table that includes all sampling sites and to expand the discussion (also just one sentence) by considering differentiation within the Mediterranean Sea and comparing them with Atlantic locations.

 Specifically, I have two questions. First, what is the authors' hypothesis regarding genetic differentiation between the Adriatic and Tyrrhenian/Western Mediterranean regions? Second, could some genetic markers (outliers) show similarities between geographically distant sampling sites in the Atlantic and Mediterranean Sea?

 As in this study, similar findings of Mediterranean genetic differences were reported by Rodríguez-Ezpeleta et al. (2016), but also that study did not explore in depth non-neutral markers of this region.

 I understand that this could be not the primary objective of the manuscript and I also recognize the potential limitations arising from the relatively low number of sampling sites and individuals in the Mediterranean, which could affect the interpretation of the results. However, some attempt to discuss or comment on the observed differences in the considered areas could be insightful.

 In this work, the authors conducted an analysis for outlier detection of the two Atlantic regions (indicating lack of sub structuring), but not for the Mediterranean—or at least, no mention of such analysis is made in the manuscript. If evidence of selective pressures related to environmental factors are found between Mediterranean populations and in relation to Atlantic ones, it could provide a highly interesting (even if not conclusive) perspective that might offer new insights into genetic similarities or differences among spawning components worldwide.

 To clarify, as noted by the authors, previous studies on other species have suggested that genetic differentiation can arise due to adaptation to specific environmental factors (such as bathymetry, salinity, dissolved oxygen….etc.). This is typically reflected in genetic markers under selective pressure (outliers). For example, in *Engraulis encrasicolus*, the same genes associated with lower salinity habitat have been shown to drive the evolution of ecotypes inhabiting estuarine areas, even in geographically distant regions like the Bay of Biscay, the Gulf of Cádiz, and the Tyrrhenian Sea (Catanese G, et al., 2017 [Ref-1]). Considering this, the authors may want to explore whether similar patterns of adaptive differentiation are present in their dataset in relation to different areas.

 In conclusion, I believe this manuscript is a valuable contribution to the field, both for its scientific rigor and its potential impact. I appreciate the authors' efforts to enhance the clarity and depth of their study and, although I had some suggestions, consider the manuscript suitable for indexing.

**Updated Note:** 

 I agree with the authors' response and I appreciate their clarification. Their explanation effectively addresses my curiosity regarding the inclusion of Mediterranean data and its role as an outgroup in the study. I also understand that further analyses on Mediterranean populations would shift the focus away from the main objectives of the research. This answer fully satisfies my inquiries.

Is the study design appropriate and does the work have academic merit?

Yes

Is the work clearly and accurately presented and does it cite the current literature?

Yes

If applicable, is the statistical analysis and its interpretation appropriate?

Yes

Are all the source data underlying the results available to ensure full reproducibility?

Yes

Are the conclusions drawn adequately supported by the results?

Partly

Are sufficient details of methods and analysis provided to allow replication by others?

Yes

Reviewer Expertise:

I mainly work on the application of molecular techniques to fisheries and aquaculture studies, investigating the genetic structure and evaluating the current status of genetic variability of wild populations of fisheries resources and marine organisms. Moreover, I work on authenticity of fishery products, on the detection of pathogens affecting marine organisms and on strategies to minimize by-catches of traditional fishing for a gradual elimination of discards in European fisheries

I confirm that I have read this submission and believe that I have an appropriate level of expertise to confirm that it is of an acceptable scientific standard.

## References

[1] : Insights on the drivers of genetic divergence in the European anchovy. *Sci Rep* .2017;7(1) : 10.1038/s41598-017-03926-z 4180 10.1038/s41598-017-03926-z 28646131 PMC5482869

# Response

**Published:** 2025-02-11

**Authors:** Naiara Rodriguez Ezpeleta

**Affiliations:** Fundacion AZTI Sukarrieta, Sukarrieta, Euskadi, Spain

We sincerely thank the reviewer for their thorough examination of our work and the positive feedback provided. While we appreciate the interest in the Mediterranean population, it is important to clarify that this is not the primary focus of our study. The Mediterranean data were included as an outgroup to improve understanding of mackerel populations on both sides of the Atlantic Ocean. Additionally, as noted by the reviewer, the sample size from the Mediterranean is limited and does not consider spawning and non-spawning individuals from various areas. Including the suggested analyses and speculating on the evolutionary history of Mediterranean populations would divert attention from the main focus of our research. The data have been made publicly available and is available at the GenBank SRA archive since 2016, allowing any interested party to analyze them independently or in conjunction with additional samples. We hope this clarification is satisfactory.

# Response

**Published:** 2025-02-18

**Authors:** GAETANO CATANESE

**Affiliations:** IRFAP LIMIA, Govern de les Illes Balears, Port d'Andratx, Spain

I agree with the authors' response and I appreciate their clarification. Their explanation effectively addresses my curiosity regarding the inclusion of Mediterranean data and its role as an outgroup in the study. I also understand that further analyses on Mediterranean populations would shift the focus away from the main objectives of the research. This answer fully satisfies my inquiries.

# Reviewer report: Reviewer response for version 2

**DOI:** 10.21956/openreseurope.20953.r50346

**Published:** 2025-02-11

**Authors:** Yusuf Bektas

**Affiliations:** 1 Faculty of Arts and Sciences, Department of Molecular Biology and Genetics, Karadeniz Technical University, Ortahisar, Turkey

Dear Editor,

 This manuscript investigates the population structure of Atlantic mackerel ( *Scomber scombrus*) by genetic analysis to investigate whether there are genetically distinct spawning components. Whole genome sequencing (WGS) and RAD-seq methods were used to test whether there are genetic differences between the Northwest Atlantic (NWA), Northeast Atlantic (NEA) and Mediterranean (MED) populations. It was determined that the Atlantic mackerel population is genetically divided into three main units and that there are no genetically distinct spawning components within NWA and NEA. Mackerel in Greenland is genetically identical to the NEA population, i.e., a northward expansion was observed. Climate change has been shown to affect the distribution of mackerel, but no genetic adaptation signals were detected. The authors confirmed these results using ADMIXTURE, PCA, FST and SNP under selection analyses. The study has important implications for population genetics, fisheries management and climate change impacts.

 The research question is clear and meaningful. The genetic structure of Atlantic mackerel and an important issue in terms of fisheries management are addressed. The sampling includes sufficient geographical coverage for population genetics. A lack of samples from Southern Europe (south of Portugal, North Africa) was noted. Additional samples from this region could have been collected to expand the analyses. The current sampling strategy can be validated by supporting it with fisheries records.

 The combination of whole genome sequencing (WGS) and RAD-seq provides a robust genetic approach.

 Population genetic analyses were appropriate and meticulously implemented. Linkage disequilibrium (LD) control was performed and appropriate SNP filtering was applied for the analyses. Statistical significance was achieved using multiple test correction (FDR). Permutation tests and statistical cross-validations were performed. Adaptive SNPs can be validated with additional selection analyses such as BAYESCAN or FLK.

 The results are consistent with the literature and scientifically sound. Although the FST values ​​are low, they are correctly interpreted as significant. The claim that Atlantic mackerel does not exhibit homing behavior is supported by genetic data. The result that the mackerels in Greenland are the same as the NEA population has been analyzed in accordance with the principles of population genetics. Could functional analyses be performed for the SNPs under selection? It can be tested whether these SNPs are associated with genetic adaptation processes.

 Potential adaptive mechanisms can be examined by determining which genes are located in the relevant genome regions. Can genetic data be integrated with ecological factors such as sea temperatures and migration routes?

 All genetic data and analysis codes have been shared as open access. The bioinformatics analysis pipeline has been documented and reproducibility has been ensured.

 In conclusion

 The study is scientifically sound, meticulously prepared and strong in terms of genetic analyses. The results are sufficiently supported by the existing data, but certain improvements can be made. It is strong in terms of reproducibility and is a transparent study thanks to the open data policy.

 Best regards,

Is the study design appropriate and does the work have academic merit?

Yes

Is the work clearly and accurately presented and does it cite the current literature?

Yes

If applicable, is the statistical analysis and its interpretation appropriate?

Yes

Are all the source data underlying the results available to ensure full reproducibility?

Yes

Are the conclusions drawn adequately supported by the results?

Yes

Are sufficient details of methods and analysis provided to allow replication by others?

Yes

Reviewer Expertise:

Phylogenetic systematic and Population Genetics

I confirm that I have read this submission and believe that I have an appropriate level of expertise to confirm that it is of an acceptable scientific standard.

# Reviewer report: Reviewer response for version 1

**DOI:** 10.21956/openreseurope.18768.r44860

**Published:** 2024-11-07

**Authors:** Julia Tovar Verba

**Affiliations:** 1 Division of Evolutionary Biology, Faculty of Biology, Ludwig-Maximilians-Universität München, Munich, Germany

In the manuscript entitled “Atlantic mackerel population structure does not support genetically distinct spawning components”, the authors aim to examine the genetic differentiation and connectivity of the species, adding to and clarifying conflicting results in previous studies. The authors utilize broadly distributed samples, an annotated reference genome and thousands of genomic markers, which are appropriate for addressing questions related to population structure of marine species, with large and highly connected populations. The authors also make a good effort in predicting future changes, and in providing clear information for management. Overall, the manuscript is clearly articulated, and the methodology is robust. However, I believe the paper can be improved by addressing some minor points.

 In the introduction, could you please include since when the historical records related to Atlantic mackerel distribution exist? This could include information about early documentation of the species distribution patterns and any observed shifts prior to recent changes - if existent. Also in the introduction, the authors mention that the whole range of the species was sampled, although as mentioned later on in the manuscript, there are no samples from Portugal or from the North Atlantic African coast. In the methods section, I would recommend a more thorough explanation regarding the inclusion of all age classes. Additionally, it is not clear if there was no dataset with only Mediterranean samples. In the results section, I recommend the integration of the K = 2 plot within the main text since it might be relevant to see the shared ancestry between the Mediterranean and Northeast Atlantic. Also, I would like to see the other Ks (4 and maybe 5) in the supplementary material, to see if the division in the Mediterranean populations appears. In the discussion, could you please clarify the sentence “In addition, the species shows high plasticity as it can tolerate up to 5-15°C (Olafsdottir et al., 2019) (ref-1) despite its optimal temperature range (9-13°C), which is possibly linked to the high genomic heterozygosity detected.”? It is hard to understand if both plasticity and heterozygosity are on the species or individual level. In the discussion, I recommend making it clear that the sample distribution of this study is more broadly distributed in comparison with other studies. Since there were previous papers with a similar objective and methods, it would be good to see it clear what is the addition of this manuscript. The discussion surrounding adaptation mechanisms is currently limited and could be improved. But mainly I would like to see a more thorough examination of Mediterranean population divergence in the discussion.

Is the study design appropriate and does the work have academic merit?

Yes

Is the work clearly and accurately presented and does it cite the current literature?

Yes

If applicable, is the statistical analysis and its interpretation appropriate?

Yes

Are all the source data underlying the results available to ensure full reproducibility?

Yes

Are the conclusions drawn adequately supported by the results?

Yes

Are sufficient details of methods and analysis provided to allow replication by others?

Yes

Reviewer Expertise:

Ecology, population genomics, marine biology

I confirm that I have read this submission and believe that I have an appropriate level of expertise to confirm that it is of an acceptable scientific standard.

## References

[1] : Geographical expansion of Northeast Atlantic mackerel (Scomber scombrus) in the Nordic Seas from 2007 to 2016 was primarily driven by stock size and constrained by low temperatures. *Deep Sea Research Part II: Topical Studies in Oceanography* .2019;159: 10.1016/j.dsr2.2018.05.023 152-168 10.1016/j.dsr2.2018.05.023

# Response

**Published:** 2025-01-09

**Authors:** Naiara Rodriguez Ezpeleta

**Affiliations:** Fundacion AZTI Sukarrieta, Sukarrieta, Euskadi, Spain

We would like to thank the reviewer for assessing our manuscript and for offering valuable insights. Below we provide answers to the comments raised:

 COMMENT: In the introduction, could you please include since when the historical records related to Atlantic mackerel distribution exist? This could include information about early documentation of the species distribution patterns and any observed shifts prior to recent changes - if existent.  

**ANSWER: We have decided not to add this information to the introduction section as we do not think it is essential for the purpose of the study.**

 COMMENT: Also in the introduction, the authors mention that the whole range of the species was sampled, although as mentioned later on in the manuscript, there are no samples from Portugal or from the North Atlantic African coast.

**ANSWER: replaced “across the distribution” by “across most of the distribution”**

 COMMENT: In the methods section, I would recommend a more thorough explanation regarding the inclusion of all age classes. 

**ANSWER: we have included the fact that adults included spawning and non-spawning individuals; details about age of each individual are included in a supplementary table.**

 COMMENT: Additionally, it is not clear if there was no dataset with only Mediterranean samples. 

**ANSWER: The purpose of this study is to assess connectivity within the Atlantic and in particular within each side of the Atlantic. This is why a dataset with only mediterranean samples was not created. Also, the Mediterranean samples included had already been analyzed in a previous study (Rodríguez-Ezpeleta et al. 2016 Molecular Ecology Resources)**

 COMMENT: In the results section, I recommend the integration of the K = 2 plot within the main text since it might be relevant to see the shared ancestry between the Mediterranean and Northeast Atlantic.

**ANSWER: We have kept K=2 in supplementary material not to overload Figure 1 which is already quite busy.**

 COMMENT: Also, I would like to see the other Ks (4 and maybe 5) in the supplementary material, to see if the division in the Mediterranean populations appears.

**ANSWER: The division between Mediterranean populations has already been assessed in a previous study (Rodriguez-Ezpeleta et al. 2016 Molecular Ecology Resources) and no new Mediterranean samples have been included in this study.**

 COMMENT: In the discussion, could you please clarify the sentence “In addition, the species shows high plasticity as it can tolerate up to 5-15°C (Olafsdottir et al., 2019) (ref-1) despite its optimal temperature range (9-13°C), which is possibly linked to the high genomic heterozygosity detected.”? It is hard to understand if both plasticity and heterozygosity are on the species or individual level.  

**ANSWER: We have added, “at the species level”**

 COMMENT: In the discussion, I recommend making it clear that the sample distribution of this study is more broadly distributed in comparison with other studies. Since there were previous papers with a similar objective and methods, it would be good to see it clear what is the addition of this manuscript. 

**ANSWER: added “and most broadly distributed samples” to the first sentence of introduction.**

 COMMENT: The discussion surrounding adaptation mechanisms is currently limited and could be improved. But mainly I would like to see a more thorough examination of Mediterranean population divergence in the discussion. 

**ANSWER: As mentioned above, the Mediterranean sea population is out of the scope of this study.**

# Reviewer report: Reviewer response for version 1

**DOI:** 10.21956/openreseurope.18768.r44524

**Published:** 2024-10-16

**Authors:** Sven Winter

**Affiliations:** 1 Senckenberg Biodiversity and Climate Research Centre (BiK-F), Frankfurt am Main, Germany; 2 University of Veterinary Medicine, Vienna, Austria

In this study, the authors sequenced, assembled, and annotated the reference genome of the Atlantic mackerel from Oxford Nanopore long-read and Illumina short-read data.

 They further used this reference to map newly generated population-level RAD-sequencing data and study population structure in the North Atlantic, aiming to identify the impact of spawning components on the genetic structure of the species and identify the origin of individuals of the recent northward expansion.

 The authors provide a vital genetic resource, which, quite frankly, I expected to be generated years ago due to the species' economic value.

 The chosen sequencing, assembly, and annotation methods are all very sound and follow well-established procedures. However, they fall just a little short of the current standards set by most genome consortia due to the lack of proximity-ligation scaffolding, which results in an assembly that is not (near) chromosome level.

 The methods used for the population genomic analyses are also sound and well-established. Yet, they do not exhaust the possibilities the dataset grants them, especially for the task set by the aims mentioned by the authors. If the task was to study spawning components, then the authors need to define those locations and analyze the genetic makeup of individuals in more detail. A standard procedure of PCA and Admixture is a nice start, but I would suggest including more analyses on genetic connectivity, gene flow, and, ideally (if the density of Rad loci permits it), structural variation, which has been shown to influence population structure and behavior of populations drastically (e.g., in quails but also scallops). In addition, even though this is a species with large numbers, I am wondering if the current state of fisheries management would benefit from analyses focused on effective population size estimates (e.g., GONE, Ne-Estimator etc.) to see if there is a genetic impact over recent decades.

 I want to congratulate the authors on generating a great dataset. I feel that the study so far is well-written and well-conducted, but it would benefit from some more in-depth analyses to support the conclusions and gain additional information useful for the management of the species.

Is the study design appropriate and does the work have academic merit?

Yes

Is the work clearly and accurately presented and does it cite the current literature?

Yes

If applicable, is the statistical analysis and its interpretation appropriate?

Partly

Are all the source data underlying the results available to ensure full reproducibility?

Yes

Are the conclusions drawn adequately supported by the results?

Partly

Are sufficient details of methods and analysis provided to allow replication by others?

Yes

Reviewer Expertise:

genome assembly, population genomics

I confirm that I have read this submission and believe that I have an appropriate level of expertise to confirm that it is of an acceptable scientific standard, however I have significant reservations, as outlined above.

# Response

**Published:** 2025-01-09

**Authors:** Naiara Rodriguez Ezpeleta

**Affiliations:** Fundacion AZTI Sukarrieta, Sukarrieta, Euskadi, Spain

We would like to thank the reviewer for the insightful comments provided on our manuscript. Below, we provide an answer to each of them:

**COMMENT: **The chosen sequencing, assembly, and annotation methods are all very sound and follow well-established procedures. However, they fall just a little short of the current standards set by most genome consortia due to the lack of proximity-ligation scaffolding, which results in an assembly that is not (near) chromosome level.

**ANSWER:** Yes, we are aware: the reason for this was the difficulty of obtaining high quality DNA from mackerel tissue samples despite using freshly caught individuals and following similar procedures than with other fish species for which we were successful. In response to this comment and to a similar one by reviewer 1, we have replaced “high-quality genome” by “genome” throughout the manuscript.

**COMMENT:** The methods used for the population genomic analyses are also sound and well-established. Yet, they do not exhaust the possibilities the dataset grants them, especially for the task set by the aims mentioned by the authors. If the task was to study spawning components, then the authors need to define those locations and analyze the genetic makeup of individuals in more detail. A standard procedure of PCA and Admixture is a nice start, but I would suggest including more analyses on genetic connectivity, gene flow, and ideally (if the density of Rad loci permits it), structural variation, which has been shown to influence population structure and behaviour of populations drastically (e.g., in quails but also scallops).

**ANSWER: **By using analyses based on outliers, we have implicitly tested presence of potential structural variants, as presence of these should reveal alternative groupings in PC analyses as we have seen in other studies (see for example Diaz-Arce et al. 2024 Molecular Ecology). Here, despite doing analyses based on outlier markers, we do not find any additional pattern suggesting a structural variant that is large enough to be detectable with the SNPs included in our study.   

**COMMENT:** In addition, even though this is a species with large numbers, I am wondering if the current state of fisheries management would benefit from analyses focused on effective population size estimates (e.g., GONE, Ne-Estimator etc.) to see if there is a genetic impact over recent decades.

**ANSWER: **The most critical question for Atlantic mackerel at the moment is that about the presence of isolated spawning components, which is the one we mostly tackle in this study. Of course, analyses looking at the effect of effective population size over time would be interesting, but we do not think that the samples we have allow for such analyses, given that we have a small number of individuals per year/location/cohort. However, we are sure that the genetic resources we have produced will be extremely valuable for other studies wishing to continue on this matter.

# Response

**Published:** 2025-01-13

**Authors:** Naiara Rodriguez Ezpeleta

**Affiliations:** Fundacion AZTI Sukarrieta, Sukarrieta, Euskadi, Spain

We would like to thank the reviewer for the insightful comments provided on our manuscript. Below, we provide an answer to each of them:  

**COMMENT:** The chosen sequencing, assembly, and annotation methods are all very sound and follow well-established procedures. However, they fall just a little short of the current standards set by most genome consortia due to the lack of proximity-ligation scaffolding, which results in an assembly that is not (near) chromosome level.  

**ANSWER: **Yes, we are aware: the reason for this was the difficulty of obtaining high quality DNA from mackerel tissue samples despite using freshly caught individuals and following similar procedures than with other fish species for which we were successful. In response to this comment and to a similar one by reviewer 1, we have replaced “high-quality genome” by “genome” throughout the manuscript.  

**COMMENT:** The methods used for the population genomic analyses are also sound and well-established. Yet, they do not exhaust the possibilities the dataset grants them, especially for the task set by the aims mentioned by the authors. If the task was to study spawning components, then the authors need to define those locations and analyze the genetic makeup of individuals in more detail. A standard procedure of PCA and Admixture is a nice start, but I would suggest including more analyses on genetic connectivity, gene flow, and ideally (if the density of Rad loci permits it), structural variation, which has been shown to influence population structure and behaviour of populations drastically (e.g., in quails but also scallops).

**ANSWER: **By using analyses based on outliers, we have implicitly tested presence of potential structural variants, as presence of these should reveal alternative groupings in PC analyses as we have seen in other studies (see for example Diaz-Arce et al. 2024 Molecular Ecology). Here, despite doing analyses based on outlier markers, we do not find any additional pattern suggesting a structural variant that is large enough to be detectable with the SNPs included in our study.

**COMMENT:** In addition, even though this is a species with large numbers, I am wondering if the current state of fisheries management would benefit from analyses focused on effective population size estimates (e.g., GONE, Ne-Estimator etc.) to see if there is a genetic impact over recent decades.

**ANSWER: **The most critical question for Atlantic mackerel at the moment is that about the presence of isolated spawning components, which is the one we mostly tackle in this study. Of course, analyses looking at the effect of effective population size over time would be interesting, but we do not think that the samples we have allow for such analyses, given that we have a small number of individuals per year/location/cohort. However, we are sure that the genetic resources we have produced will be extremely valuable for other studies wishing to continue on this matter.

# Reviewer report: Reviewer response for version 1

**DOI:** 10.21956/openreseurope.18768.r40457

**Published:** 2024-06-07

**Authors:** Natalia Bayona

**Affiliations:** 1 Division of Natural Science and Mathematics, Oxford College, Emory University, Oxford, Georgia, USA

In the manuscript entitled “Atlantic Mackerel Population Structure Does Not Support Genetically Distinct Spawning Components,” the authors aim to sequence, assemble, and annotate the whole genome of *Scomber scombrus* and use such to map restriction-enzyme associated fragments to call SNPs from individuals across most of the current geographic distribution range. These methods were used to assess contrasting hypotheses of either fine genetic differences (based on otolith, meristic, allozymes, mitochondrial, and microsatellite markers) or mixing (based on tagging and a recent genomic study) between spawning components present within each of the recognized populations/stocks of the species (NEA, NWA, and MED). The authors also aimed to identify genetic markers associated with local adaptation processes of these spawning groups. Finally, researchers assessed the origin of the population expansion that species suffer northwards and the potential loci associated with this shift.

 I applaud the researchers' work and effort in generating a brand-new genome assembly and annotation for the species. This is a valuable resource that is long due for this and other marine species with high economic and ecological relevance in their distribution range. I found the methods and results regarding genome assembly and annotation sounding and appropriate. Minor suggestions are given below.

 In terms of the population genetics analyses, the methods used are also suitable for the system. However, in terms of the hypotheses laid in the introduction, and thus the ones I believe the authors aim to address, I have some recommendations. First, the authors, in the title and introduction, state that they aim to assess if spawning components correspond to genetic discrete or mixed groups. However, the study falls short in delineating the currently recognized spawning components, assigning the samples collected to these components, and explicitly considering these in the data analyses. For example, in the Northeast Atlantic, there have been recognized three components in the spawning seasons (Southern: Spain and Portugal, Western: Bay of Biscay and Ireland, and North Sea; Jansen and Gislason., 2013; Collette and Graves 2019). In the Northwest Atlantic, two distinct spawning components, northern: Canada-Gulf of St. Lawrence, and southern: US-Mid-Atlantic Bight to Gulf of Main, have been proposed (Moura et al., 2020). This study has the potential to directly test for genetic differentiation between spawning components if samples are grouped and assigned accordingly for analyses. I understand that given the results from ADMIXTURE and PCAs, there seems to be no relevant signal, and it is likely that the pattern will be maintained, but to make strong conclusions (included in the title), specific analyses between spawning components should be performed. For example, if the color or shape in the PCAs could represent the spawning components rather than (or additionally to) sampling locations, there could be a more conspicuous and straightforward signal (lack of), and the conclusion would be more obvious. Similarly, it is interesting that FST values are very low for different stocks within the Mediterranean. If the significance of these values can be included, along with those for comparisons of components in reference samples, that would also aid the audience in understanding the evidence of differences/similarities between spawning components. Also, under this context, it may be relevant for the authors to acknowledge that no samples are obtained from what would represent the spawning Southern NEA component, and thus, conclusions should be taken carefully given this underrepresentation. Finally, I would recommend authors assess if there are fine-scale differences within spawning seasons (years), given that they collected samples that span across multiple years (2011-2017) in different components and the grouping of all of these just by location, regardless of timing, may cofound genetic effects may have within seasons. While doing this, it is important to also omit individuals from the respective spawning areas that were not in the process of spawning (including only certain spawning adults; if larvae are included, it is important to maybe segregate by season).

 Additionally, other studies have argued population connectivity between the western Mediterranean (Barcelona) and the NEA (Zardoya et al., 2004), which is not evident in this study even though the westernmost sample from the Mediterranean (WESTMED) is even closer to the Atlantic than the sample from Zardoya et al., 2004. This is valuable evidence that should be highlighted in the discussion.

 Overall, the study has a good set of analyses and presents and discusses trends in a logical and pertinent fashion. I celebrate the effort and great amount of work and thought the authors have placed into the study. My main concern was highlighted above, and minor suggestions and comments on the study can be found below, which include some suggestions in scientific writing as well.

Major comments:

  

1. Figure S2 is identical to Figure 3 from Cruz et al., 2023, Refer ref [4]. Although the two papers have one co-author in common, in this case, I suggest the authors remove the figure from the supplementary and instead provide proper attributions to the published paper.
2. The title and introduction of the paper suggest that the core analyses rely on detecting whether spawning components are genetically different. However, such comparisons are not presented in methods or results. For example, the colors and shapes of the PCA and ADMIXTURE analyses correspond to geographically distinct locations but not to spawning components. In my letter above, I presented examples of how to consider the established spawning components in the analyses and the year of the samples.

Minor comments:

*Title*

 “Atlantic mackerel population structure does not support genetically distinct spawning components.” It is a strong title, but conclusions should be drawn from explicit analyses, suggested in major comments.

*Abstract*

  

1. The background information could be enhanced by including the current understanding of the number of populations and spawning components that have been characterized in the distribution range.
2. I would not consider this a “high-quality” genome assembly.

*Intro*

1. I would add to the first sentence that the species is **coastal** pelagic so that geographic distribution breaks in the North Atlantic can be more easily understood.
2. I would add “… Labrador, **Canada** to Cape Lookout, **United States** in the west.” If countries are given for the eastern population instead of cities, it would be good to maintain some consistency.
3. It is relevant that the authors highlight that the species is also present in the Black Sea.
4. When citing the spawning groups in the Northwest, it is important that authors recognize the original work of O’Brien et al. 1993.
5. When citing the spawning groups in the Northeast, it is important that authors recognize the original work of Nesbø et al., 1999-2000.
6. I celebrate the initiative of the authors to generate a reference genome for the species, but I would not call this assembly a "high-quality" given the large number of scaffolds still present.
7. The authors portray one of the objectives to assess the origin of the Greenlandic/Islandic population and markers associated with the recent northward migration. I found it relevant here to cite hypotheses based on Jansen et al., 2016.

*Materials & Methods*

  

1. I suggest the authors differentiate in this section the samples used for genome assembly and annotation from the samples used for population analyses. For example, it is unclear which sample was used for genome sequencing. Was it the same female used for its annotation? If so, what tissue was used for DNA extraction and sequencing? Where was the female captured? Also, no further information is given regarding the amount and quality of DNA used for library preparation.
2. I recommend that authors include either in Figure 2A or in a summary table the groups or samples representing the spawning components and even maybe stages and years of collection per locality. The latter is because, in the introduction, the authors make the case that other studies did not account for differences between year classes/generations, which limits the scope of conclusions. Although this information is present in Supplemental Materials, it should be easy to access and summarized by the authors in this section so the audience can grasp the power of these analyses.
3. When using Guppy for base calling, which mode/parameters were used? For example, was the mode “high-accuracy” used for both data types?
4. The authors claimed, “The free alternative open-source high performance base calling software Dorado can also be used to base call the reads”. Do they mean that they use this software? If so, for which reads and what version?
5. Remove the extra SbfI in the fourth line of the third paragraph of methods and include that the enzyme is at 10 U/uL.
6. Correct typo “Enzime” for “Enzyme” on line six of the third paragraph of methods.
7. If the authors did not use the 10X T4 DNA ligase buffer from ThermoFisher, but an NEB buffer instead, please provide the catalog number from NEB of the buffer used.
8. The ATP typically used for libraries is at 10 mM, but here authors state that they used a 100 mM, could you please provide manufacturer and catalogue number?
9. What were the number of cycles and times for the shearing of DNA fragments in Covaris?
10. Correct typo “1,5” for “1.5” on line 18 of the first paragraph of page 6.
11. Revise that throughout the text, spaces should separate numbers and their units. There is inconsistency throughout.
12. In the second line of the second paragraph of page six, the 5 uL is not per sample but per ligated pool, right? Also, please mention the primers used here, including the manufacturer. Revise spaces in the PCR profile.
13. The document containing Fig S1 and S2 is not named “Extended Data” but “Supplementary Material.”
14. Please include the versions of some software under “Genome Assembly and Annotation”: TrimGalore, Fitlong2, BWA, HyPo, purge_dups, etc.
15. What version of Piccard was used for deduplication?
16. Missing a parenthesis closure when explaining the data set used to assess connectivity between spawning components.
17. Please include versions of the ADMIXTURE and R packages used.
18. Please specify what subset or subsets of individuals were used for pcadapat and its version.

*Results*

1. Table 2 has a column title “SCO1A Annotation” However, the genome assembly label is fScoSco3.1, what does SCO1A means in this context?
2. There is one minor discrepancy between Table 1 and the ENA project information according to the accession number provided by the authors. Table 1 states a total number of sequences of 4,483, and ENA states 4,482. The total length in the table is 741,290,963 bp and in ENA 741,290,950 bp.
3. The use of GenomeScope for genome profiling included in FigS3 should be included in the methods and properly cited.
4. In table S2, the thousandth separators under the column contigs should be a comma and not a quote symbol. Also, the “Coverage” column should indicate units, which I believe are “X.”
5. The title of Figure S4 should say that the colors match those from Figure 2, not 1.
6. Revise writing of sentence “FST values further validated this split, as all pairwise comparisons between the three groups resulted **high** with the **highest** FST value reported for MED vs NWA (FST=0.035)”. Also, the NEA vs MED should be FST=0.019 instead of 0.19.
7. The authors mentioned in the methods that the statistical significance of Fst values is assessed and adjusted, but these values are not presented in the results. Please include these values. They are particularly relevant for the Fst values presented within the Mediterranean Sea.
8. Remove the close parenthesis in the sentence: “This differentiation within the Mediterranean Sea is also seen in the FST pairwise comparisons, with FST 0.003 and 0.004 for the Adriatic Sea versus the Tyrrhenian Sea and Western Mediterranean Sea, respectively **)**.”

*Discussion*

1. When discussing the origin of the Greenlandic/Islandic population, it is relevant to cite the original work of Jansen et al., 2016.
2. In the paragraph: “Despite inter-population FST values being low (FST=0.014-0.035), these are in the range of what is usually observed in marine fish species with large population sizes since FST estimates are affected by both effective population sizes and migration rates, which may derive into insufficient statistical power provided by neutral markers to reject the panmictic hypothesis” I am not sure if this is arguing that the values observed are not statistically significant. Please clarify and make sure to include the p-values of the tests in the results.
3. The paragraph: “Furthermore, the high rate of heterozygosity detected at genome level may reflect the presence of a large metapopulation, for which patterns of low diversity would persist even in the presence of uneven migration along its distribution… ” results are confusing to me. High values of heterozygosity indicate high and not low genetic diversity. What the cited study argues is that in populations (not an individual), high levels of heterozygosity may be associated with large effective population sizes at a metapopulation level rather than very high local effective population sizes, but migration helps homogenize these values in the metapopulation. To further address this, the authors of this study should then present the values of heterozygosity observed globally and locally with the SNPs and address such hypotheses rather than making such relevant final remarks based on the heterozygosity of one individual for which the origin was not depicted. Finally, to draw conclusions on metapopulation models, I recommend that the authors test such hypotheses, perhaps by estimating the effective number of migrants between spawning components and the three established populations.

I congratulate the authors for the hard work they have put in so far, and I believe that with the proper focus/direction, the manuscript will be able to be indexed in the near future.

 Thanks for allowing me to review the manuscript.

Is the study design appropriate and does the work have academic merit?

Yes

Is the work clearly and accurately presented and does it cite the current literature?

Yes

If applicable, is the statistical analysis and its interpretation appropriate?

Partly

Are all the source data underlying the results available to ensure full reproducibility?

Yes

Are the conclusions drawn adequately supported by the results?

Partly

Are sufficient details of methods and analysis provided to allow replication by others?

Yes

Reviewer Expertise:

Genomics, bioinformatics, population genetics, conservation genetics

I confirm that I have read this submission and believe that I have an appropriate level of expertise to confirm that it is of an acceptable scientific standard, however I have significant reservations, as outlined above.

## References

[1] : Population structure of Atlantic mackerel (Scomber scombrus). *PLoS One* .2013;8(5) : 10.1371/journal.pone.0064744 e64744 10.1371/journal.pone.0064744 23741381 PMC3669354

[2] : Population structure and dynamics of the Atlantic mackerel (Scomber scombrus) in the North Atlantic inferred from otolith chemical and shape signatures. *Fisheries Research* .2020;230: 10.1016/j.fishres.2020.105621 10.1016/j.fishres.2020.105621

[3] : Differential population structuring of two closely related fish species, the mackerel (Scomber scombrus) and the chub mackerel (Scomber japonicus), in the Mediterranean Sea. *Mol Ecol* .2004;13(7) : 10.1111/j.1365-294X.2004.02198.x 1785-98 10.1111/j.1365-294X.2004.02198.x 15189203

[4] : Chromosome-level assembly and annotation of the Xyrichtys novacula (Linnaeus, 1758) genome. *DNA Res* .2023;30(5) : 10.1093/dnares/dsad021 10.1093/dnares/dsad021 37797305 PMC10590160

[5] : Phylogeography and population history of Atlantic mackerel (Scomber scombrus L.): a genealogical approach reveals genetic structuring among the eastern Atlantic stocks. *Proc Biol Sci* .2000;267(1440) : 10.1098/rspb.2000.0998 281-92 10.1098/rspb.2000.0998 10714883 PMC1690521

[6] : Ocean warming expands habitat of a rich natural resource and benefits a national economy. *Ecol Appl* .2016;26(7) : 10.1002/eap.1384 2021-2032 10.1002/eap.1384 27755730

[7] : Tunas and Billfishes of the World. *Johns Hopkins University Press:Baltimore, Maryland* .2019;

[8] : Maturation of nineteen species of finfish off the northeast coast of the United States, 1985-1990. *National Oceanographic and Atmospheric Administration (NOAA)* .1993;

# Response

**Published:** 2025-01-13

**Authors:** Naiara Rodriguez Ezpeleta

**Affiliations:** Fundacion AZTI Sukarrieta, Sukarrieta, Euskadi, Spain

We would like to thank the reviewer for the insightful comments to our work. Please find below the comments provided and corresponding answers.  

 COMMENT: In terms of the population genetics analyses, the methods used are also suitable for the system. However, in terms of the hypotheses laid in the introduction, and thus the ones I believe the authors aim to address, I have some recommendations. First, the authors, in the title and introduction, state that they aim to assess if spawning components correspond to genetic discrete or mixed groups. However, the study falls short in delineating the currently recognized spawning components, assigning the samples collected to these components, and explicitly considering these in the data analyses. For example, in the Northeast Atlantic, there have been recognized three components in the spawning seasons (Southern: Spain and Portugal, Western: Bay of Biscay and Ireland, and North Sea; Jansen and Gislason., 2013; Collette and Graves 2019). In the Northwest Atlantic, two distinct spawning components, northern: Canada-Gulf of St. Lawrence, and southern: US-Mid-Atlantic Bight to Gulf of Main, have been proposed (Moura et al., 2020). This study has the potential to directly test for genetic differentiation between spawning components if samples are grouped and assigned accordingly for analyses. I understand that given the results from ADMIXTURE and PCAs, there seems to be no relevant signal, and it is likely that the pattern will be maintained, but to make strong conclusions (included in the title), specific analyses between spawning components should be performed. For example, if the colour or shape in the PCAs could represent the spawning components rather than (or additionally to) sampling locations, there could be a more conspicuous and straightforward signal (lack of), and the conclusion would be more obvious.

**ANSWER: Larvae, juveniles and spawning adults are the samples representing spawning components; thus instead of coloring by spawning locations, which are not easy to clearly delimit, we have labelled samples according to age class also in the Figures 2D and E to better show the lack of differentiation, even when considering only reference samples corresponding to potential spawning components.   **  

 COMMENT: Similarly, it is interesting that FST values are very low for different stocks within the Mediterranean. If the significance of these values can be included, along with those for comparisons of components in reference samples, that would also aid the audience in understanding the evidence of differences/similarities between spawning components. **ANSWER: P-values of pairwise FST between Mediterranean locations and Atlantic reference samples comparisons now are included in the text (results section).**  

 COMMENT: Also, under this context, it may be relevant for the authors to acknowledge that no samples are obtained from what would represent the spawning Southern NEA component, and thus, conclusions should be taken carefully given this underrepresentation.

**ANSWER: Lack of comprehensive sampling of the southern NEA locations consequences of this gap are disclosed and commented in the Discussion section.**  

 COMMENT: Finally, I would recommend authors assess if there are fine-scale differences within spawning seasons (years), given that they collected samples that span across multiple years (2011-2017) in different components and the grouping of all of these just by location, regardless of timing, may cofound genetic effects may have within seasons. While doing this, it is important to also omit individuals from the respective spawning areas that were not in the process of spawning (including only certain spawning adults; if larvae are included, it is important to maybe segregate by season).

**ANSWER: Analysis of differentiation between reference samples spanning different years and across different locations warrant the same results of no differentiation between spawning components, both at individual based level ( see ADMIXTURE and PCA analysis in Fig. 2) as well as for Fst inferences as shown below (one group (CPCOD2015L) had to be removed from pop-based inference as only one larva was captured in that location/year.** **NEA (FST\p-values)** **PCPNE2013L (42)** **CANTB2013L (42)** **PCPNE2013SA (17)** **BBISC2013** **SA (21)** **CANTB2013SA (27)** **NOSEA2017SA (21)** **PCPNE2013L (42)** **0,449** **0,000** **0,000** **0,023** **0,002** **CANTB2013L (42)** **0,000** **0,000** **0,000** **0,003** **0,000** **PCPNE2013SA (17)** **0,002** **0,001** **0,000** **0,000** **0,001** **BBISC2013SA (21)** **0,001** **0,001** **0,000** **0,000** **0,001** **CANTB2013SA (27)** **0,000** **0,000** **0,000** **0,000** **0,000** **NOSEA2017SA (21)** **0,001** **0,001** **0,001** **0,001** **0,000** **NWA (FST\p-values)** **CPCOD2004L (6)** **CPCOD2013L (7)** **CPCOD2016L (17)** **STLAW2012SA (28)** **CPCOD2004L (6)** **-** **0,086** **1,000** **1,000** **CPCOD2013L (7)** **0,002** **-** **1,000** **1,000** **CPCOD2016L (17)** **0,000** **0,000** **-** **1,000** **STLAW2012SA (28)** **0,000** **0,000** **0,000** **-**    

 COMMENT: Additionally, other studies have argued population connectivity between the western Mediterranean (Barcelona) and the NEA (Zardoya et al., 2004), which is not evident in this study even though the westernmost sample from the Mediterranean (WESTMED) is even closer to the Atlantic than the sample from Zardoya et al., 2004. This is valuable evidence that should be highlighted in the discussion.

**ANSWER: Reference Zardoya et al., 2004 has been added to the discussion section; however, we have decided not to put more emphasis on this because our study focuses on the Atlantic spawning components and because we already showed and discussed this in a previous work (Rodriguez-Ezpeleta et al. 2016).**

 COMMENT: Figure S2 is identical to Figure 3 from Cruz et al., 2023, Refer ref [4]. Although the two papers have one co-author in common, in this case, I suggest the authors remove the figure from the supplementary and instead provide proper attributions to the published paper. **ANSWER: This figure highlights the workflow followed for genome assembly as support for the methodological approach. Our work has no connection with the work of Cruz et al. 2023 other than the fact that both studies used the same standard workflow for genome assembly (as many others); thus, we have decided to maintain the figure as Supplementary Material.**  

 COMMENT: The title and introduction of the paper suggest that the core analyses rely on detecting whether spawning components are genetically different. However, such comparisons are not presented in methods or results. For example, the colors and shapes of the PCA and ADMIXTURE analyses correspond to geographically distinct locations but not to spawning components. In my letter above, I presented examples of how to consider the established spawning components in the analyses and the year of the samples **.**

**ANSWER: Our analyses do show comparisons among components because they use reference samples labelled by both location and age class. In order to make this clearer, as requested by the reviewer above, the main figure has been modified accordingly.**  

 COMMENT: Title - “Atlantic mackerel population structure does not support genetically distinct spawning components.” It is a strong title, but conclusions should be drawn from explicit analyses, suggested in major comments.

**ANSWER: We believe that our analyses strongly support what the title states. We have used reference samples (larvae, juveniles and spawning adults) from the different recognized spawning locations and no differentiation is seen between them. This implies that spawning components are not genetically distinct. **  

 COMMENT: Abstract - The background information could be enhanced by including the current understanding of the number of populations and spawning components that have been characterized in the distribution range.

**ANSWER: We believe that the information currently in the abstract is sufficient**  

 COMMENT: I would not consider this a “high-quality” genome assembly.

**ANSWER: “High-quality” was removed as suggested. **  

 COMMENT: Intro I would add to the first sentence that the species is coastal pelagic so that geographic distribution breaks in the North Atlantic can be more easily understood.

**ANSWER: Done**  

 COMMENT: I would add “… Labrador, Canada to Cape Lookout, United States in the west.” If countries are given for the eastern population instead of cities, it would be good to maintain some consistency.

**ANSWER: Done**  

 COMMENT: It is relevant that the authors highlight that the species is also present in the Black Sea.

**ANSWER: Done**  

 COMMENT: When citing the spawning groups in the Northwest, it is important that authors recognize the original work of O’Brien et al. 1993. ANSWER: Done   COMMENT: When citing the spawning groups in the Northeast, it is important that authors recognize the original work of Nesbø et al., 1999-2000.

**ANSWER: Done**  

 COMMENT: I celebrate the initiative of the authors to generate a reference genome for the species, but I would not call this assembly a "high-quality" given the large number of scaffolds still present.

**ANSWER: we replaced “high-quality genome” by “genome” throughout the text**  

 COMMENT: The authors portray one of the objectives to assess the origin of the Greenlandic/Islandic population and markers associated with the recent northward migration. I found it relevant here to cite hypotheses based on Jansen et al., 2016.

**ANSWER: mentioned and cited.**  

 COMMENT: Materials & Methods - I suggest the authors differentiate in this section the samples used for genome assembly and annotation from the samples used for population analyses. For example, it is unclear which sample was used for genome sequencing. Was it the same female used for its annotation? If so, what tissue was used for DNA extraction and sequencing? Where was the female captured? Also, no further information is given regarding the amount and quality of DNA used for library preparation.  

**ANSWER: Information on tissue type and quantity for samples used for population structure is already specified in the section “Tissue sampling and nucleotide extraction” and in “Library preparation and sequencing”. Information on the sample used for assembly and annotation are now included in “Tissue sampling and nucleotide extraction”.  **  

 COMMENT: Materials & Methods - I recommend that authors include either in Figure 2A or in a summary table the groups or samples representing the spawning components and even maybe stages and years of collection per locality. The latter is because, in the introduction, the authors make the case that other studies did not account for differences between year classes/generations, which limits the scope of conclusions. Although this information is present in Supplemental Materials, it should be easy to access and summarized by the authors in this section so the audience can grasp the power of these analyses.

**ANWER: This information is already included in the supplementary material as the reviewer stated and we do not see the need to include a summary table as part of the main manuscript**  

 COMMENT: Materials & Methods - When using Guppy for base calling, which mode/parameters were used? For example, was the mode “high-accuracy” used for both data types?

**ANSWER: Parameters used are now reported in the text.**  

 COMMENT: Materials & Methods - The authors claimed, “The free alternative open-source high performance base calling software Dorado can also be used to base call the reads”. Do they mean that they use this software? If so, for which reads and what version?

**ANSWER: This software was not used, but it is a request from Ocean Research Europe (ORC journal) to provide free available alternative to replicate analyses in the case the program used as a paying wall, as in our case. Thus, the software was included as a comparable suggestion to the one we used. **  

 COMMENT: Materials & Methods - Remove the extra SbfI in the fourth line of the third paragraph of methods and include that the enzyme is at 10 U/uL.

**ANSWER: done**  

 COMMENT: Materials & Methods - Correct typo “Enzime” for “Enzyme” on line six of the third paragraph of methods.

**ANSWER: done**  

 COMMENT: Materials & Methods - If the authors did not use the 10X T4 DNA ligase buffer from ThermoFisher, but an NEB buffer instead, please provide the catalog number from NEB of the buffer used.  

**ANSWER: done**  

 COMMENT: Materials & Methods - The ATP typically used for libraries is at 10 mM, but here authors state that they used a 100 mM, could you please provide manufacturer and catalogue number?

**ANSWER: done**  

 COMMENT: Materials & Methods - What were the number of cycles and times for the shearing of DNA fragments in Covaris?

**ANSWER: done**  

 COMMENT: Materials & Methods - Correct typo “1,5” for “1.5” on line 18 of the first paragraph of page 6.

**ANSWER: done**  

 COMMENT: Materials & Methods - Revise that throughout the text, spaces should separate numbers and their units. There is inconsistency throughout.  

**ANSWER: Done**  

 COMMENT: Materials & Methods - In the second line of the second paragraph of page six, the 5 uL is not per sample but per ligated pool, right? Also, please mention the primers used here, including the manufacturer. Revise spaces in the PCR profile.

**ANSWER: After samples are pooled, all reagents used are reported per ligated pool. Primers and PCR profile have been included.**  

 COMMENT: Materials & Methods - The document containing Fig S1 and S2 is not named “Extended Data” but “Supplementary Material.”  

**ANSWER: Document has been renamed**  

 COMMENT: Materials & Methods - Please include the versions of some software under “Genome Assembly and Annotation”: TrimGalore, Fitlong2, BWA, HyPo, purge_dups, etc. **ANSWER: Done**  

 COMMENT: Materials & Methods - What version of Piccard was used for deduplication? **ANSWER: Done**  

 COMMENT: Materials & Methods - Missing a parenthesis closure when explaining the data set used to assess connectivity between spawning components.  

**ANSWER: corrected**  

 COMMENT: Materials & Methods - Please include versions of the ADMIXTURE and R packages used.  

**ANSWER: Done**  

 COMMENT: Materials & Methods - Please specify what subset or subsets of individuals were used for pcadapat and its version.  

**ANSWER: Revised**  

 COMMENT: Results - Table 2 has a column title “SCO1A Annotation” However, the genome assembly label is fScoSco3.1, what does SCO1A means in this context?

**ANSWER: Removed SCO1A and left only Annotation**  

 COMMENT: Results - There is one minor discrepancy between Table 1 and the ENA project information according to the accession number provided by the authors. Table 1 states a total number of sequences of 4,483, and ENA states 4,482. The total length in the table is 741,290,963 bp and in ENA 741,290,950 bp.

**ANSWER: We thank the reviewer for highlighting the discrepancy. The correct stats are the ones in the ENA, thus Table 1 now reports those values. The difference is due to a 13 nt sequence that was removed when preparing the final assembly files.**  

 COMMENT: Results - The use of GenomeScope for genome profiling included in FigS3 should be included in the methods and properly cited.

**ANSWER: done**  

 COMMENT: Results - In table S2, the thousandth separators under the column contigs should be a comma and not a quote symbol. Also, the “Coverage” column should indicate units, which I believe are “X.”

**ANSWER: changed**  

 COMMENT: Results - The title of Figure S4 should say that the colors match those from Figure 2, not 1.

**ANSWER: changed**  

 COMMENT: Results - Revise writing of sentence “FST values further validated this split, as all pairwise comparisons between the three groups resulted high with the highest FST value reported for MED vs NWA (FST=0.035)”. Also, the NEA vs MED should be FST=0.019 instead of 0.19.

**ANSWER: revised**  

 COMMENT: Results - The authors mentioned in the methods that the statistical significance of Fst values is assessed and adjusted, but these values are not presented in the results. Please include these values. They are particularly relevant for the Fst values presented within the Mediterranean Sea.

**ANSWER:  P-values of all Fst pairwise comparisons are now included in the Results as well as in Supplementary Tables. **  

 COMMENT: Results - Remove the close parenthesis in the sentence: “This differentiation within the Mediterranean Sea is also seen in the FST pairwise comparisons, with FST 0.003 and 0.004 for the Adriatic Sea versus the Tyrrhenian Sea and Western Mediterranean Sea, respectively).”

**ANSWER: done**  

 COMMENT: Discussion - When discussing the origin of the Greenlandic/Islandic population, it is relevant to cite the original work of Jansen et al., 2016.

**ANSWER: work cited**  

 COMMENT: Discussion - In the paragraph: “Despite inter-population FST values being low (FST=0.014-0.035), these are in the range of what is usually observed in marine fish species with large population sizes since FST estimates are affected by both effective population sizes and migration rates, which may derive into insufficient statistical power provided by neutral markers to reject the panmictic hypothesis” I am not sure if this is arguing that the values observed are not statistically significant. Please clarify and make sure to include the p-values of the tests in the results.

**ANSWER: All p-values are now included in the results. **  

 COMMENT: Discussion -The paragraph: “Furthermore, the high rate of heterozygosity detected at genome level may reflect the presence of a large metapopulation, for which patterns of low diversity would persist even in the presence of uneven migration along its distribution…” results are confusing to me. High values of heterozygosity indicate high and not low genetic diversity. What the cited study argues is that in populations (not an individual), high levels of heterozygosity may be associated with large effective population sizes at a metapopulation level rather than very high local effective population sizes, but migration helps homogenize these values in the metapopulation. To further address this, the authors of this study should then present the values of heterozygosity observed globally and locally with the SNPs and address such hypotheses rather than making such relevant final remarks based on the heterozygosity of one individual for which the origin was not depicted. Finally, to draw conclusions on metapopulation models, I recommend that the authors test such hypotheses, perhaps by estimating the effective number of migrants between spawning components and the three established populations.

**ANSWER: We have reworked this sentence to accommodate the reviewer´s comment.**
